# A multiplex implantable microdevice assay identifies synergistic combinations of cancer immunotherapies and conventional drugs

**DOI:** 10.1038/s41587-022-01379-y

**Published:** 2022-07-04

**Authors:** Zuzana Tatarova, Dylan C. Blumberg, James E. Korkola, Laura M. Heiser, John L. Muschler, Pepper J. Schedin, Sebastian W. Ahn, Gordon B. Mills, Lisa M. Coussens, Oliver Jonas, Joe W. Gray

**Affiliations:** 1 Department of Biomedical Engineering, OHSU Center for Spatial Systems Biomedicine, Portland, OR USA; 2 Knight Cancer Institute, Oregon Health & Science University, Portland, OR USA; 3 Department of Radiology, Brigham & Women’s Hospital, Harvard Medical School, Boston, MA USA; 4 Department of Cell, Developmental and Cancer Biology, Oregon Health & Science University, Portland, OR USA; 5 Division of Oncologic Sciences, Oregon Health & Science University, Portland, OR USA

**Keywords:** Targeted therapies, Assay systems, Breast cancer, Complexity, Cancer immunotherapy

## Abstract

Systematically identifying synergistic combinations of targeted agents and immunotherapies for cancer treatments remains difficult. In this study, we integrated high-throughput and high-content techniques—an implantable microdevice to administer multiple drugs into different sites in tumors at nanodoses and multiplexed imaging of tumor microenvironmental states—to investigate the tumor cell and immunological response signatures to different treatment regimens. Using a mouse model of breast cancer, we identified effective combinations from among numerous agents within days. In vivo studies in three immunocompetent mammary carcinoma models demonstrated that the predicted combinations synergistically increased therapeutic efficacy. We identified at least five promising treatment strategies, of which the panobinostat, venetoclax and anti-CD40 triple therapy was the most effective in inducing complete tumor remission across models. Successful drug combinations increased spatial association of cancer stem cells with dendritic cells during immunogenic cell death, suggesting this as an important mechanism of action in long-term breast cancer control.

## Abstract (web summary)

Testing of combinations of cancer immunotherapies and conventional drugs yields promising leads.

## Main

Modern cancer therapies increasingly seek to effect tumor control by simultaneously attacking tumor-intrinsic vulnerabilities, enhancing anti-tumor immune activity and/or mitigating stromal mediators of resistance. Targeted drugs typically are designed to attack genetic or transcriptional vulnerabilities on which tumor cells depend for survival but non-malignant cells do not^1^. Genomic screening approaches have supported such tumor-intrinsic aspects of precision medicine, leading to matching of genomic aberrations with specific targeted agents^2^. In breast cancer, treatments targeting tumors that depend on estrogen receptor (ER) signaling, aberrant signaling resulting from human epidermal growth factor receptor 2 (HER2) amplification and/or overexpression, CDK4/6 signaling and defects in DNA repair in triple-negative breast cancer (TNBC) have been particularly effective^3^. Unfortunately, these treatments are not uniformly effective even in primary tumors carrying the target and are usually only transiently effective in metastatic disease^4,5^. This may be due, in part, to drug modulation of aspects of the tumor microenvironment (TME), including immune function, that negatively influence cancer control. This suggests that treatment efficacy can be increased by combining these drugs with agents that increase immunogenicity and/or counter microenvironment-mediated resistance, a hypothesis that we address in this paper.

The concept of enhancing cancer treatment efficacy by combining chemotherapies and targeted drugs with agents that enhance immune-mediated anti-tumor activity is increasingly well-established^6^. The clearest example is the use of immunotherapies, including immune checkpoint blocking (ICB) antibodies as complements to tumor-targeted therapies in various liquid and solid malignancies^7^. However, many cancers do not benefit from ICB, including in breast cancer where efficacy has been limited to a subset of patients with TNBC^8,9^. This lack of efficacy has been attributed, in part, to two mechanisms: (1) low antigenicity through decreased expression of major histocompatibility complex class I (MHC-I) proteins, observed mainly in luminal ER^+^ breast cancer^4,10^ and HER2^+^ breast cancer^11,12^; and (2) a naturally immunosuppressive TME associated mainly with TNBC and HER2^+^ breast cancer^13,14^. Both of these mechanisms may limit CD8^+^ T-cell-mediated anti-tumor responses that then cannot be leveraged to improve efficacy of ICB therapies^15^. Combinations of conventional chemotherapies and/or targeted anti-cancer drugs that increase immunogenic cell killing promise substantial improvements in overall outcome^16,17^. However, further understanding of drug–immune system interactions is required to design effective and safe immune-modulating combinatorial regimens.

A variety of experimental approaches have been deployed to elucidate the effects of drug combinations on the tumor and stromal components and to identify biomarkers that inform on the efficacy of treatment combination decisions^1^. Biomarkers typically are identified by establishing associations between tumor features and outcomes in clinical studies^18^, such as those supported by the National Cancer Institute National Clinical Trials Network^19^, The Cancer Genome Atlas^20^ and Human Tumor Atlas Network^21^ programs. However, these association-based approaches need to be tested for causality in systems that faithfully recreate the interactions of the various components of the TME. Common model systems include tumors that arise in patient-derived xenografts (PDXs) and immune competent mice and short-term or long-term ex vivo cultures comprised of tumor and stromal components using organoid systems, miniscule scaffolds and/or active fluidics to closely model specific aspects of the TME^22,23^. However, the whole animal mouse studies typically are slow, expensive and labor-intensive, and comprehensive modeling and faithful recapitulation of all TME interactions, especially of the immune component, in ex vivo and PDX systems remains a major challenge^1^.

We report here on an integrated in vivo approach to rapidly, safely and efficiently assess the effects of multi-drug treatments on the TME composition and architecture in living mice. Our study focuses on mouse mammary cancers, and our approach is based on the intratumor delivery of nanoliter doses (nanodoses) of multiple drugs or drug combinations into spatially separate regions of a tumor using a minimally invasive, implantable microdevice (IMD)^24–26^ and multiplexed immunohistochemical (mIHC) assessments^27,28^ of the in situ responses of the TME milieu near each drug delivery site. Computational analyses of serial mIHC staining and imaging of more than 30 proteins allow precise characterization of tumor cell states (for example, proliferation, stemness, antigenicity and cell death) as well as comprehensive classification of cells comprising the TME, including immune cells, vasculature and other stroma cells. Assessment of the composition and spatial distribution of the functionally different cell types in each drug delivery area facilitates identification of drug-mediated mechanisms of response and resistance that suggest new therapeutic interventions. We refer to this approach as the Multiplex Implantable Microdevice Assay (MIMA), and we used it to evaluate the effects of five targeted anti-cancer agents (olaparib, palbociclib, venetoclax, panobinostat and lenvatinib) and two chemotherapies (doxorubicin and paclitaxel) to predict synergistic anti-tumor effects with different immune-based therapies. The data predicted that palbociclib would synergize with anti-CSF1R, venetoclax with anti-CD40 and panobinostat with anti-PD-1 immunotherapy, respectively, which we validated in traditional systemic dosing studies. We found the triple combination of panobinostat, venetoclax and anti-CD40 to be curative and well-tolerated across multiple models of mammary cancer. We suggest immunogenic cell death and spatial association of licensed dendritic cells (DCs) with cancer stem cells (CSCs) as the likely mechanism underlying CSC-specific anti-tumor immunity in breast cancer for long-term tumor control.

## Results

### MIMA components and design

**Fig. 1:**
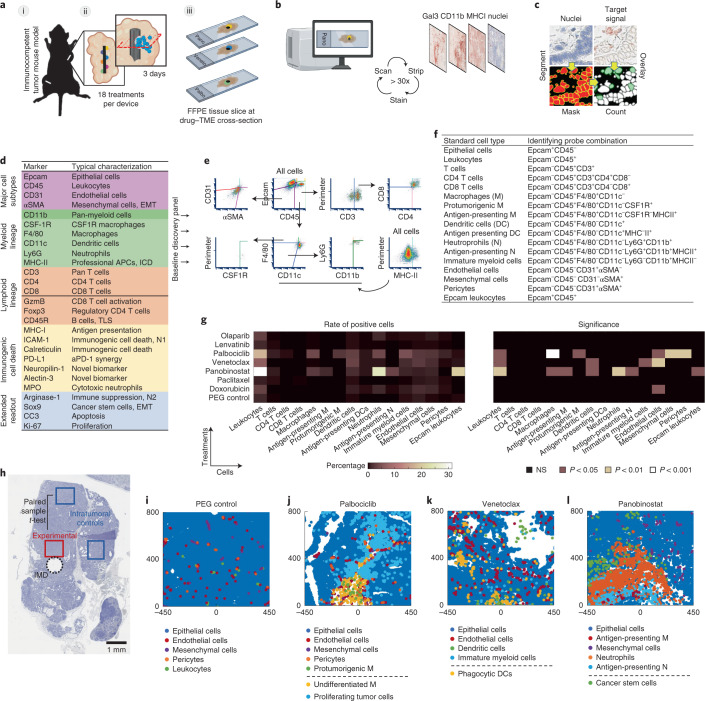
MIMA components and testing of locally induced drug effects on TME. **a**, Schematic of IMDs implanted into a multifocal mouse model of mammary carcinoma (i) showing treatments being released into spatially separated regions of tumors through passive diffusion (ii) and each condition being assayed individually at an angle perpendicular to the device (ii and iii). **b**, Schematic of the mIHC technique composed of iterative histological stripping, staining and scanning using digital scanning microscopy to detect the target set of markers. **c**, Acquired images are co-registered with nuclear staining, and the mean intensity of antibody staining within a mask is calculated for each cell to count marker-positive cells in a spatially intact tissue. **d**, Antibody list primary probe classification used to interrogate a broad range of tumor-intrinsic and TME states. **e**,**f**, Multidimensionality reduction in hierarchical gating (**e**) and list of probe combinations identifying standard cell types (**f**). **g**,**h**, Heat map of mean percentage of positive cells (left) and level of significance (right) at depicted targeted agents and chemotherapies (*y* axis), with PEG being the negative control (**g**). Total cell counts were between 3,000 and 5,000 cells per assay area and were matched ±300 total cells for paired samples: experimental versus control region as shown in the macroscopic view of the hematoxylin-stained tumor tissue implanted with IMD (black dashed circle; **h**). Minimum population proportion within 5% margin of error and 95% confidence level was set to 0.75% (represents 12 cells) to discriminate noise from specific signal. *n* = 3 wells from three tumors from 2–3 mice per treatment. Significance was calculated by paired sample one-tailed *t*-test. MMTV-PyMT mice with late-stage spontaneously growing tumors were implanted for 3 days. **i**–**l**, Presentation of selected standard cell types in *x*–*y* space. [0,0] coordinate is the drug-releasing site; direction of release is upward. Schematics in **a**,**b** were partly generated with BioRender. NS, not significant.

**Extended Data Fig. 1:**
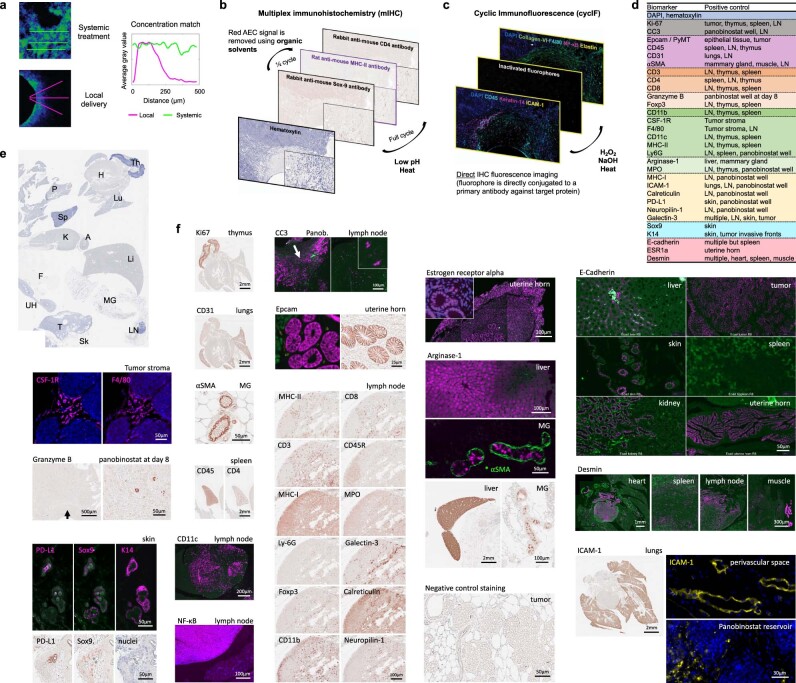
Components of the MIMA system and mIHC/cycIF anti-mouse antibody validation. **a**, Comparison of systemic and local drug dosing using implantable microdevice (IMD; adapted and modified from (Jonas et al., 2015)). Sample images of intratumoral doxorubicin distribution at 6 hours after systemic treatment (top left) and PEG-formulated doxorubicin transport from device at 20 hours after release (bottom left). Signal mean intensity was extracted, averaged (*n* = 3 each) and plotted using a moving average window filter. For detailed information on the pharmacokinetics of intratumoral drug release from the IMD see (Jonas et al., 2015). **b**,**c** Schematic overview of multiplex immunohistochemistry (mIHC; **b**) and cyclic immunofluorescence (cycIF, **c**). **b**, mIHC utilizes indirect staining, iterative deposition of chromogen/enzyme pairs and brightfield microscopy to image the target signal. The chromogen used in this study is called 3-amino-9ethylcarbazol (AEC) and it produces a red precipitate when visualized. AEC is susceptible to organic solvents which is used to remove the red signal and detect two target proteins in one cycle. Primary antibody mixture is stripped in heated low pH citrate buffer is every cycle after scanning in order to further multiplex the staining on a single FFPE slide. Antibodies raised in rabbit and rat hosts alternate to prevent crosstalk between cycles. Hematoxylin counterstains nuclei to allow cell count and downstream image analysis (Extended Data Fig. 2). **c**, cycIF utilizes fluorophores as reporters via direct labeling. Four to five non-overlapping fluorescent signals can be detected in a single cycle against dark background. DAPI signal is used to visualize nuclei for cell count. Progressive staining is enabled by inactivating the fluorophore using base hydrogen peroxide mixture and heat. Antibody specificity is cross-validated by performing chromogenic mIHC on the adjacent tissue section. **d**, List of biomarkers (left column) and positive control tissues used for antibody validation and signal thresholding. **e**, Hematoxylin staining of an FFPE section containing all positive control organs from an adult wild type FVB/N female mouse: thymus (Th), heart (H), lungs (Lu), liver (Li), mammary gland (MG), lymph node (LN), spleen (Sp), pancreas (P), adrenal gland (A), kidney (K), fat (F), uterine horn (UH). Tumor (T) with implanted device and attached skin (Sk) was embedded into the same FFPE block. **f**, Representative images of stated biomarkers using mIHC (red signal in bright field images) or cycIF (magenta signal in fluorescent images unless otherwise stated). Biomarker name is located on the top left; while the name of the organ is located on the top right side of each image, respectively. Some positive signal can be detected in a macroscopic view (Ki67, CD31, CD4, CD45, NF-KB, desmin, arginase-1, ICAM-1). Section stained without primary antibody served as negative control in the mIHC procedure (last image). Green fluorescent channel served to detect autofluorescence and to separate background from specific staining in the cycIF procedure. Only antibodies with very strong specific staining such as aSMA (marked with a star *), were used in conjugation with Alexa fluor-488. Scale bars; shown.

The IMD used for drug delivery in the MIMA system was a 5-mm-long, 0.75-mm-diameter biocompatible resin cylinder that delivered multiple drugs or drug combinations in up to 18 spatially separate regions inside a living tissue (Fig. 1a). IMDs were loaded with drugs formulated with polyethylene glycol (PEG) in semi-solid form so that drugs are released with controlled kinetics upon implantation via passive diffusion^24^. Local concentrations of drugs in the IMD were tuned to produce drug levels at each site in the tissue that recapitulate those achieved during systemic treatment (Extended Data Fig. 1a and Supplementary Table 1). Notably, the nanodoses of drugs do not generate the whole animal toxicities typically associated with systemic treatments^24^, thereby reducing the trauma that accompanies drug development.

**Extended Data Fig. 2:**
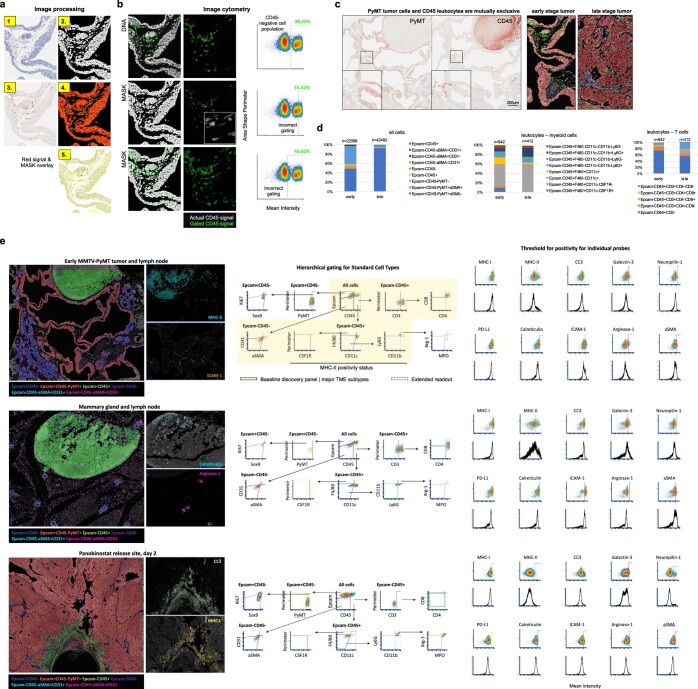
Analytical design to quantify single cell events in MIMA. **a**, Image processing for image cytometry analysis composed of the following steps, briefly: hematoxylin staining (1) is colordeconvoluted and the signal is segmented using ImageJ watershed function (Schneider et al., 2012) to generate mask (2). Red AEC signal (3) mean intensity in a selection as defined by mask (4) is calculated for each cell (5). **b**, Pixel intensity measurements and shape size measurements are used to gate cells for positive marker expression (CD45 here). FCS Express 6 and 7 Image Cytometry Software (De Novo Software), was used to obtain accurate thresholding using the cell population shape and dimensions. Accurate gating strategy is also monitored through visual inspection (second column). **c**, Tissue section of an early MMTV-PyMT mammary carcinoma and adjacent lymph node was used to establish hierarchical gating strategies in image cytometry (in E) to define “standard cell types”. This for two reasons: presence of a lymph node in the same section offers the possibility to utilize mutual exclusivity (left) for reproducible signal thresholding. Second, early tumors provide with the opportunity to evaluate broader range of phenotypically distinct cell types as compared to late-stage tumors (right and d). **d**, Percentage of positive cells in early (<0.5 cm in longest dimension) and late stage (>1.5 cm in longest dimension) MMTV-PyMT tumors. Number of cells analyzed is shown; data is derived from one and two tumors for early and late tumor sample, respectively. **e**, Density plot of dimensionality reduction in hierarchical clustering to define “standard cell types”. The shape of the gates was designed to obtain quantitatively reproducible multiplex data, batch to batch, independent of the condition measured: early tumor and lymph node (top row), mammary gland and lymph node (middle row) and panobinostat implanted tumor sample two days post exposure (bottom row) are shown for comparison. For probes other than “standard cell types” (pleiotropic/undefined biology), threshold for positivity was determined manually using FCS Express 6 and 7 Image Cytometry software (**b**) and positive control tissue (Extended Data Fig. 1d–e). Sample pictures for marker positive cells; left.

After treatment for 3 days, tumors were harvested with the IMD in place and prepared as formalin fixed, paraffin embedded (FFPE) samples, and serial tissue sections were cut orthogonal to the axis of the IMD (Fig. 1a). Sections through each drug delivery well were stained using mIHC—a process of serial immunostaining, imaging and stripping (Fig. 1b and Extended Data Fig. 1b,c)^27,28^—to assess local drug effects using a range of markers with specific staining patterns being cross-validated against those generated using cyclic immunofluorescence (cycIF)^27^ (Extended Data Fig. 1c–f). The mIHC-generated multiprotein images were analyzed by segmenting individual cells and calculating protein expression levels in each segmented cell (Fig. 1c and Extended Data Fig. 2). We developed a comprehensive mouse-specific readout panel for these studies including more than 30 proteins (Fig. 1d and Supplementary Tables 2 and 3) to interrogate a broad range of tumor and TME states and functions and identify actionable phenotypes with preferential detection of early and local responses. We selected 13 proteins (Epcam, CD45, CD31, αSMA, CD3, CD4, CD8, CD11b, F4/80, CSF1R, CD11c, Ly6G and MHC-II; Fig. 1d, baseline discovery panel) to classify 17 ‘standard cell types’ that were necessary and sufficient to capture major TME states predicting effective treatment combinations (Fig. 1e–g and Supplementary Table 4). We interrogated additional proteins to refine the 17 standard cell types and/or report on basic drug sensitivity (proliferation and apoptosis), immunogenic cell death and/or cells and processes typically associated with resistance, including CSCs (Fig. 1d, extended readout).

### MIMA identifies drug-specific histological signatures of TME response predicting rational treatment combinations

We used the MIMA system to perform a small-scale in situ screen and quantitatively assess responses to seven US Food and Drug Administration (FDA)-approved drugs with distinct modes of action. The targeted drugs were the poly (adenosine diphosphate (ADP)) ribose polymerase (PARP) inhibitor olaparib; the multi-kinase vascular endothelial growth factor receptor (VEGFR)-1/2/3 inhibitor lenvatinib; the cyclin-dependent kinase (CDK)-4/6 inhibitor palbociclib; the B cell lymphoma (BCL)-2 inhibitor venetoclax; and the pan-histone deacetylase (HDAC) inhibitor panobinostat. The chemotherapeutic drugs were the DNA-intercalating agent doxorubicin and the mitotic inhibitor paclitaxel, which are often used in first-line therapy for breast cancer^29^. We assessed the responses in tumors arising in immunocompetent MMTV-PyMT (mouse mammary tumor virus-polyoma middle tumor antigen) mice—a commonly used genetically engineered mouse model for breast cancer that mirrors many aspects of human breast cancer progression and heterogeneity^30,31^. These tumors initially express ER strongly, but expression decreases as they progress to late-stage carcinoma^31^. Gene expression profiling reveals that tumors cluster with the luminal B subtype at the stage used herein^31,32^. However, at even later stages, expression of the androgen expression increases, and the tumor may eventually model aspects of luminal androgen receptor tumors^33^. We chose a spontaneous rather than a transplanted tumor model to better account for all stages of immune-biology associated with de novo tumor progression^34^, including editing^35^.

Our MIMA analyses focused on the cell and molecular compositions and organizations that were significantly enriched in regions close to the drug delivery sites compared to distant intratumoral controls in the same tumor (Fig. 1h). The changes observed in the 17 standard cell types are summarized in Fig. 1g for all seven drugs, and Fig. 1i–l shows computed images of selected cell types after treatment.

**Extended Data Fig. 3:**
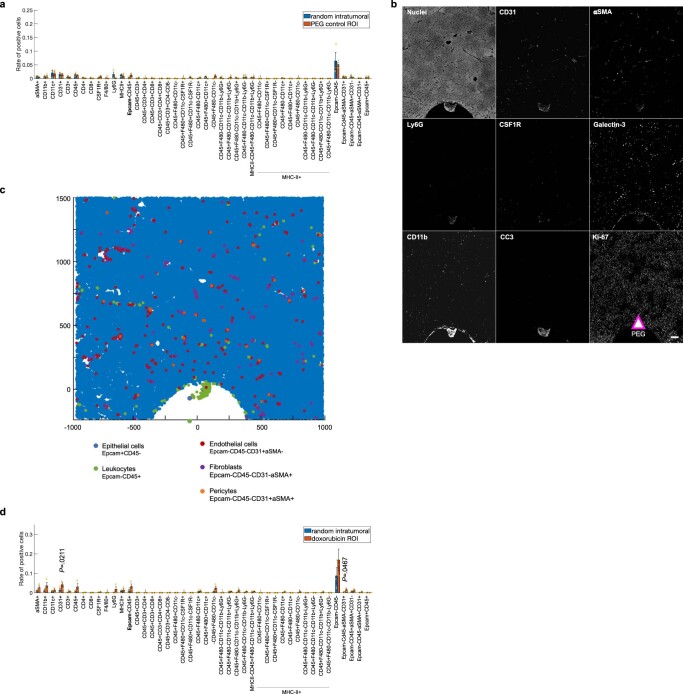
Locally induced TME changes at the PEG negative control and doxorubicin delivery sites. **a**,**d** Quantification of single cell events using individual markers and marker combinations (including standard cell types). Total cell counts to define percentage of positivity were between 3,000 to 5,000 cells per assay area and were matched ± 300 total cells for paired samples (experimental vs control region). Minimum population proportion within 5% margin of error and 95% confidence level was set to 0.75% (represents 12 cells) to discriminate noise from specific signal. Bars are mean ± s.e.; *n* = 3 reservoirs from two to three tumors. MMTV-PyMT mice with late stage (d93-d100) spontaneously growing tumors implanted with IMD for three days were used. Significance was calculated by paired sample one tailed *t-*test. **b**, Gray scale single channel images of depicted markers at the PEG-delivery reservoir. Arrow indicates the source and direction of PEG release. Scale bar; 100 µm. **c**, Presentation of main response cell types (biomarker combination displayed) in XY space. Coordinate [0,0] identifies the drug source. The direction of the drug release is upward.

Lenvatinib and paclitaxel produced no detectable effects, and they resembled those produced by the PEG negative control (Fig. 1g,i and Extended Data Fig. 3a–c), whereas olaparib caused only a modest increase in macrophage, neutrophil and fibroblast number (Fig. 1g). Doxorubicin did not mediate immune changes but did cause a significant enrichment of endothelial cells (Fig. 1g and Extended Data Fig. 3d), suggesting that normalization of vasculature^36,37^ could increase efficacy of doxorubicin in breast cancer. Palbociclib, venetoclax and panobinostat produced the strongest changes in the immune and non-immune stromal states (Fig. 1g,j,k,l). We extended mIHC analytics and performed spatial cell measurements to describe the mechanism of action of these drugs in more detail.

### Palbociclib induces enrichment of CSF1R^+^ macrophages associated with pericyte branching and de novo tumor proliferation

**Fig. 2:**
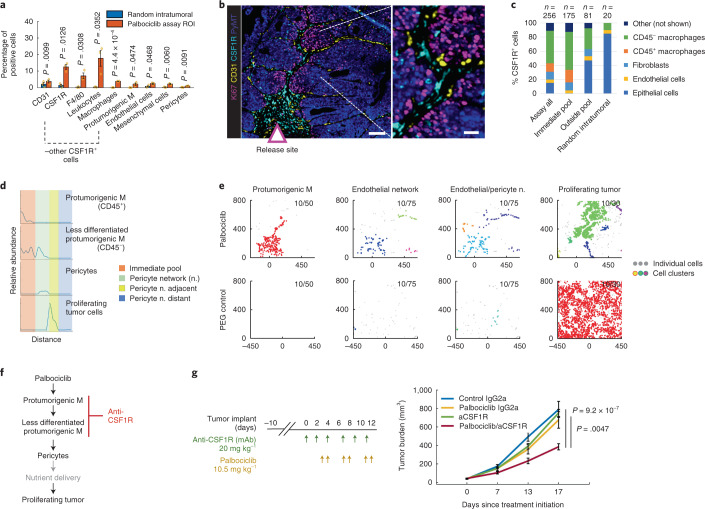
Local TME changes induced by palbociclib and whole animal studies testing the combination efficacy with predicted anti-CSF1R immunotherapy. **a**, Quantification of single-cell events using individual markers and standard cell type classification. Bars are mean ± s.e.m.; *n* = 3 reservoirs. Significance was calculated by paired sample one-tailed *t*-test. For quantification of all TME lineages, see Extended Data Fig. 4a. **b**, Sample composite image of the key response markers at the palbociclib well. Scale bars, 100 μm (left) and 25 μm (right). **c**, Percentage of top five cell types expressing CSF1R stratified by zones in the palbociclib assay area. ‘Immediate pool’ zone is visualized by the dashed line in Extended Data Fig. 4c. The number of cells analyzed (*n*) is shown. **d**, Line profile of relative cell abundance as a function of distance from well (left to right). Assay zones are color-coded in the legend; profile line is shown in Extended Data Fig. 4c. **e**, Distance-based clustering of depicted cell types as a set of *x*–*y* coordinates. Coordinate [0,0] identifies the drug source. The direction of the drug release is upward. Clusters were identified by a minimum of ten cells within maximum distances of 50 μm, 75 μm and 30 μm for CSF1R^+^ pro-tumorigenic macrophages, endothelial/pericyte network and proliferating tumor cells, respectively. Each cluster is depicted with a randomized color; individual (non-clustering) cells are shown as light gray points. **f**, Palbociclib model of response presented as line diagram and site of intervention using immunotherapy depicted in red. **g**, Tumor burden measurement of mice bearing EMT6 tumors after systemic treatment using drugs as color-coded in the graph. Shown is mean ± s.e.m.; *n* = 8–10 tumors per group. Significance was calculated using an independent two-sample, two-tailed *t*-test with equal variance. Treatment dose and schedule are presented. mAb, monoclonal antibody; ROI, region of interest.

**Extended Data Fig. 4:**
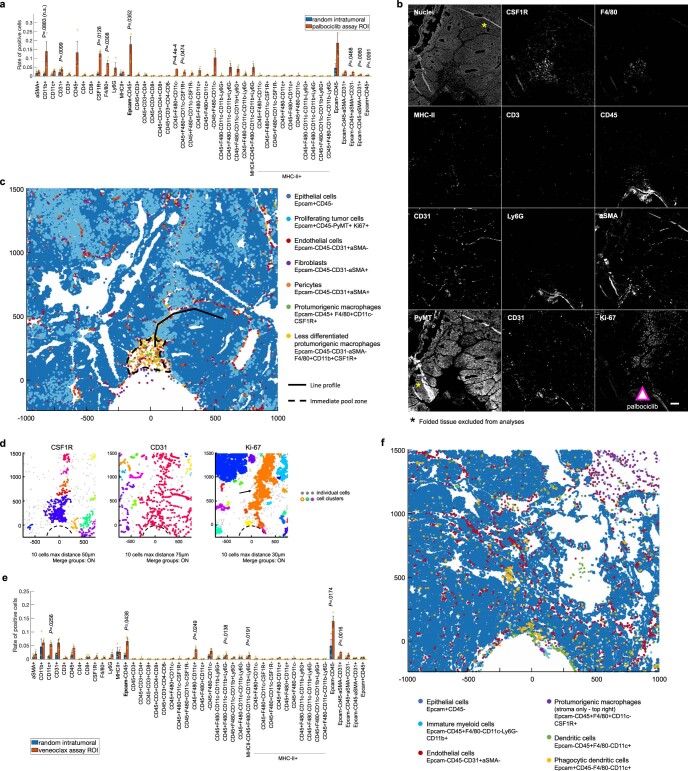
Locally induced TME changes at the palbociclib and venetoclax delivery sites. **a**,**e** Quantification of single cell events using individual markers and marker combinations (including standard cell types). Bars are mean ± s.e.; *n* = 3 reservoirs from two to three tumors. MMTV-PyMT mice with late stage spontaneously growing tumors implanted with IMD for three days were used. Significance was calculated by paired sample one tailed *t-*test. **b**, Gray scale single channel images of depicted markers at the palbociclib-delivery reservoir. Arrow indicates the source and direction of drug release. Scale bar; 100 µm. **c**,**f** Presentation of key response cell types (biomarker combination displayed) in XY space. **d**, Distance based clustering of CSF1R, CD31 and Ki67 positive cells as a set of XY coordinates. Individual clusters were identified by minimum 10 cells within maximum distance 50 µm, 75 µm and 30 µm, respectively. Clusters were merged together if present within the maximum distance range. Coordinate [0,0] identifies the drug source. Direction of the drug release is upwards.

Intratumoral treatment with palbociclib induced a significant accumulation of several stromal cell types in the assay area including CSF1R^+^, MHC-II^−^ pro-tumorigenic macrophages^6^, endothelial cells, pericytes and mesenchymal cells (Figs. 1g,j and 2a,b and Extended Data Fig. 4a–c). Spatial analyses measuring relative abundance of cells at increasing distances from the drug delivery well showed that, whereas the CD45^+^ macrophages, as classified by standard cell type, were localized to regions immediately proximal to the drug delivery well, the CD45^−^ less-differentiated macrophages^38,39^ were localized both proximally and more distally (Fig. 2c,d) and, in some regions, were associated with contractile pericytes^37^ (Fig. 2d). We also assessed the propensity of specific cell types to cluster together by mapping the locations where ten or more cells of a defined phenotype occurred together in regions 30 μm, 50 μm or 75 μm in diameter (Fig. 2e and Extended Data Fig. 4d). These analyses showed that the CSF1R^+^ macrophages and CD31^+^ endothelial cell/pericyte structures were organized together in response to the palbociclib drug stimulus and did not appear in PEG control tissues (Fig. 2e). The patterns for the CD31^+^ cell aggregates were branch-like with pericytes integrated within endothelial structures suggestive of large vessel formation and enhanced blood flow/pressure control^37^ (Fig. 2e and Extended Data Fig. 4d). The profile plot and distance-based cluster analyses also showed clusters of Ki67^+^ neoplastic cells distant from the drug delivery site and proximal to the macrophage–pericyte networks (Fig. 2d,e and Extended Data Fig. 4b,d), indicating that the macrophage–pericyte structures likely contribute to an increase in tumor cell proliferation in local microculture as summarized schematically in Fig. 2f. These results show how specific changes in TME states induced by monotherapy may mediate acquired resistance. The high expression of CSF1R on multiple cell types (Fig. 2c) and the associated increase in Ki67^+^ tumor cells (Fig. 2d,e) suggested to us that targeting the CSF1/CSF1R axis might enhance palbociclib efficacy by countering CSF1R-mediated processes (Supplementary Table 4).

We tested the performance of this MIMA prediction in a systemic treatment of the EMT6 breast cancer model, by treating mice bearing tumors orthotopically implanted into the mammary fat pads of immunocompetent syngeneic mice with intraperitoneal injections of palbociclib, an anti-CSF1R antibody monotherapy and a combination of the two. The individual drugs did not affect the rate of tumor growth. However, the combination treatment significantly reduced tumor growth (Fig. 2g). Thus, the efficacy of palbociclib/anti-CSF1R, as suggested by analyses of responses to intratumoral treatments, was confirmed in whole animal experiments.

### Venetoclax recruits phenotypically distinct clusters of DCs, immature myeloid cells and endothelial cells

**Fig. 3:**
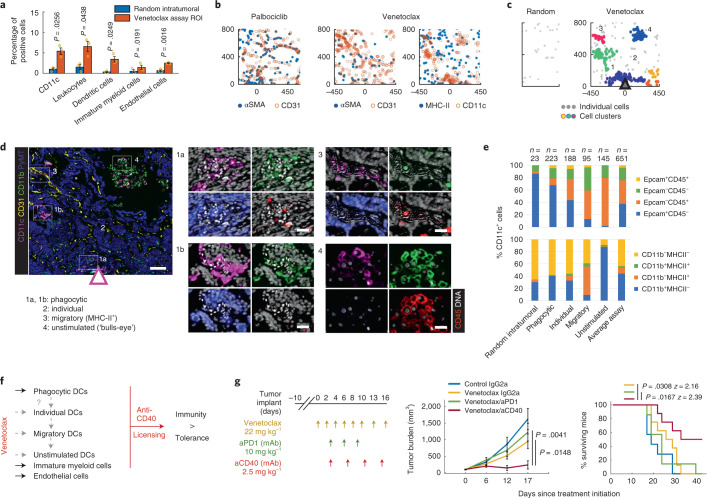
Local TME changes induced by venetoclax and whole animal studies testing the combination treatment efficacy with the predicted anti-CD40 immunotherapy. **a**, Quantification of single-cell events using individual markers and standard cell types. Bars are mean ± s.e.m.; *n* = 3 reservoirs. Significance was calculated by paired sample one-tailed *t*-test. For quantification of all cells, see Extended Data Fig. 4e. **b**, Marker co-expression in *x*–*y* coordinates in the palbociclib (left) and venetoclax (middle, right) assay areas. Each color-coded dot represents a marker-positive cell. Coordinate [0,0] identifies the drug source. The direction of the drug release is upward. **c**, Distance-based cluster analysis of CD11c^+^ cells as a set of *x*–*y* coordinates in random intratumoral (left) and venetoclax assay (right) regions. Clusters are displayed in randomized colors if at least ten cells are present within a maximum distance range of 50 μm; individual cells not meeting this criterion are shown as light gray points. **d**, Sample composite image of the key response markers at the venetoclax well. Arrow indicates the source and direction of the drug release. Numbered hashed boxes define the magnified area on the right where individual markers are overlaid on the DNA signal (in white). Scale bars, 100 μm (left) and 30 μm (right). The drug source and direction are presented by a triangle (**c**,**d**). **e**, Percentages of Epcam^+^ and CD45^+^ (top) and CD11b^+^ and MHC-II^+^ (bottom) cells within morphologically different CD11c^+^ DCs presented as a stack bar graph. The number of cells analyzed (*n*) is shown. Two to three regions of interest from two venetoclax samples were summed per each zone. **f**, Venetoclax model of response presented as an influence diagram. The drug induces recruitment of functionally different DCs, immature myeloid cells and enrichment of endothelial cells. Licensing the former two using an anti-CD40 agonist antibody shifts the balance from immune tolerance to anti-tumor immunity. Whether the different DC subsets evolve from one another or they are recruited as spatially separate entities remains to be determined (gray dashed arrows). **g**, Tumor burden measurements (left) and survival rates (right) of mice bearing E0771 tumors after systemic treatment using drugs as color-coded in the line graphs. Shown is mean ± s.e.m.; *n* = 7–8 mice per group. Significance was calculated by an unpaired two-tailed *t*-test with equal variance and by log-rank (Mantel–Cox) test for tumor burden rate and survival rate, respectively. Treatment dose and schedule are presented. For results using anti-PD-1 and anti-CD40 monotherapy, see Fig. 6c. mAb, monoclonal antibody.

Intratumor treatment with venetoclax resulted in significant recruitment of CD11c^+^ DCs, immature myeloid cells and CD31^+^ endothelial cells to the drug assay area (Figs. 1g,k and 3a and Extended Data Fig. 4e,f). Unlike in the palbociclib condition, the CD31^+^ endothelial cells did not express αSMA, suggesting that they formed small blood vessels that were not supported by pericytes^37^ (Fig. 3b). CD11c^+^ DCs, which play a critical role in regulating the balance between immune tolerance and activity^40^, were aggregated into multiple, spatially separate clusters in regions near venetoclax delivery but not in random intratumoral regions far from the drug-releasing site (Fig. 3c). The clusters differed in phenotypes defined by morphology (Fig. 3d) and expression of Epcam, CD45, MHC-II and CD11b (Fig. 3e) with distance from the reservoir. DCs closer to the reservoir exhibited brighter and smaller nuclei (Fig. 3d, regions 1a, 1b), and more than 60% were Epcam^+^CD45^−^ (Fig. 3e), suggesting that they were phagocytic^41^; whereas others displayed a ‘bull’s-eye’ membrane CD45 staining pattern typical of unstimulated myeloid cells^41^ (Fig. 3d, region 4). However, only a small fraction of these cells, which were spatially associated with endothelial cells (Fig. 3d, region 3), were MHC-II^+^ (Fig. 3e) and, thus overall, the recruited DCs were likely limited in their ability to present available tumor antigens^42^. Agonist monoclonal anti-CD40 antibodies can act on DCs and immature myeloid cells to increase their antigen-presenting capacity, maturation and activation potential (called licensing), thereby shifting the balance from tolerance to anti-tumor immunity^40,43,44^. We reasoned that this immunotherapy could be used to enhance anti-tumor capacity of the immune cells recruited by venetoclax, which were already primed to have anti-tumor activity (Fig. 3f).

Our test of this hypothesis by systemic treatment of the E0771 orthotopic breast cancer model with a combination of venetoclax and an anti-CD40 agonist showed that this combination reduced tumor growth rate and increased overall survival with 60% of mice surviving for >12 months (Fig. 3g). For comparison, the combination of venetoclax with a programmed death ligand-1 (PD-1) inhibitory antibody did not significantly affect tumor growth rate or survival (Fig. 3g). Again, a therapeutic strategy predicted by the MIMA proved to be effective in whole animal experiments.

### Panobinostat induces immunogenic cell death associated with recruitment of antigen-presenting neutrophils and macrophages

**Fig. 4:**
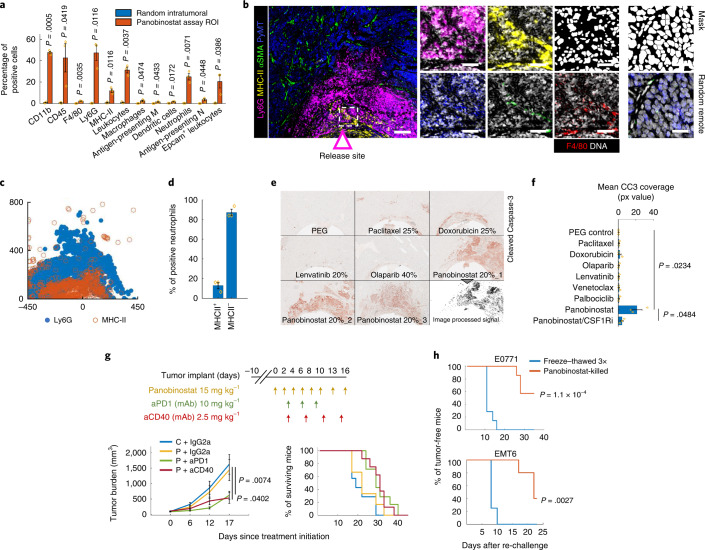
Local effects of panobinostat and whole animal studies testing induction of anti-tumor immunity in mouse mammary carcinoma. **a**, Quantification of single-cell events using individual markers and standard cell types. Bars are mean ± s.e.m.; *n* = 3 reservoirs. Significance was calculated by paired sample one-tailed *t*-test. For quantification of all cells, see Extended Data Fig. 5a. **b**, Sample composite image of the key response markers at the panobinostat well. A dashed box defines the magnified area (right), which shows F4/80 staining in red and DNA signal and DNA-derived mask in white. Scale bars, 100 μm (left) and 25 μm (right). **c**, Marker co-expression in *x*–*y* coordinates. Each dot represents a marker-positive cell. Coordinate [0,0] identifies the drug source. The direction of the drug release is upwards. **d**, Percentage of MHC-II^+^ and MHC-II^−^ neutrophils. Shown is mean ± s.e.m.; *n* = 3 panobinostat reservoirs. **e**, CC3 immunohistochemistry image of a sectioned tissue surrounding the IMD at depicted targeted agents and chemotherapies. Three replicates are presented for the most potent death-inducing drug, panobinostat. A computationally processed CC3 signal is shown as a binary image. **f**, Quantification of average mean CC3 coverage (px value) in the assay region. The graph shows mean ± s.e.m.; *n* = 3 wells per treatment; significance was calculated using an independent two-sample *t*-test with equal variance. **g**, Tumor burden measurements (left) and survival rates (right) of mice bearing E0771 tumors after systemic treatment using control diluent (C), panobinostat (P), anti-PD1, anti-CD40 and IgG2a isotype control monoclonal antibody. Shown is mean ± s.e.m.; *n* = 7–8 mice per group. Significance was calculated by an unpaired two-tailed *t*-test with equal variance and by log-rank (Mantel–Cox) test for tumor burden rate and survival rate, respectively. For results using anti-PD-1 and anti-CD40 monotherapy, see Fig. 6c. Treatment dose and schedule are presented. **h**, Induction of anti-tumor immunity measured in a vaccination study using panobinostat-treated cells and negative control (cells killed by three freeze–thaw cycles). Line graphs show percentages of mice free from palpable tumors. The *P* value was calculated by log-rank (Mantel–Cox) test. *n* = 7 per each group for E0771 model and *n* = 4 (control) and *n* = 5 (experimental) for EMT6 model, respectively. mAb, monoclonal antibody.

**Extended Data Fig. 5:**
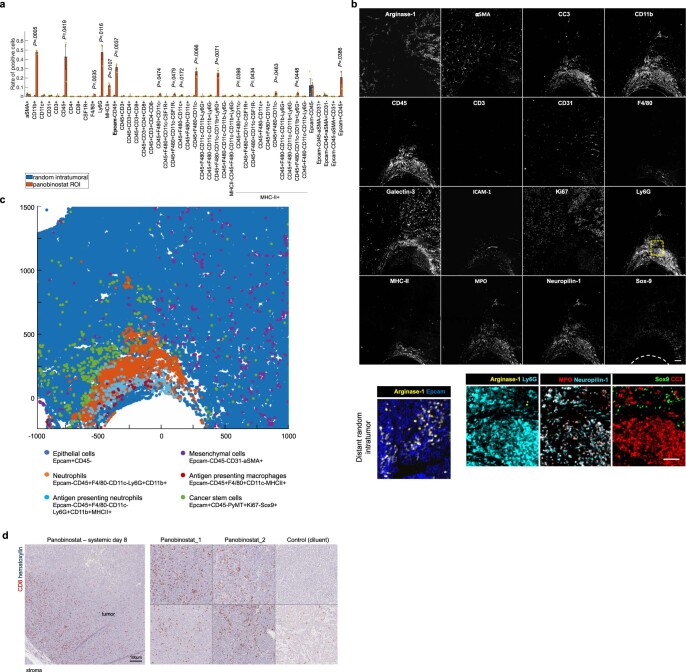
Locally induced TME changes at the panobinostat delivery site. **a**, Quantification of single cell events using individual markers and marker combinations (including standard cell types). Bars are mean ± s.e.; *n* = 3 reservoirs from two tumors from two mice. MMTV-PyMT mice with late stage spontaneously growing tumors implanted with IMD for three days were used. Significance was calculated by paired sample one tailed *t-*test. **b**, Gray scale single channel images of depicted markers at the panobinsotat-delivery reservoir. Magnified area (bottom) is shown by yellow rectangle. Hashed line shows the IMD border. Scale bar; 100 µm. **c**, Presentation of key response cell types (biomarker combination displayed) in XY space. Coordinate [0,0] identifies the drug source. The direction of the drug release is upward. **d**, CD8 (red AEC signal) and hematoxylin (blue) staining of tumors from ErbB2deltaEx16 mice treated with diluent (control) and panobinostat systemically for seven days. The experiment was repeated twice with similar results. Scale bar, 100 µm.

Intratumor delivery of panobinostat led to significant recruitment of several immune cell populations, including DCs, antigen-presenting macrophages and (antigen-presenting) neutrophils, with the latter being the most abundant (Figs. 1g,l and 4a,b and Extended Data Fig. 5a–c). Neutrophils are considered to be rapid responders against pathogens and classically are not categorized as professional antigen-presenting cells (APCs) as compared to DCs, B cells, monocytes and macrophages, which have superior ability to prime naive T cells^42^. However, 13% of neutrophils were MHC-II^+^ (Fig. 4c,d), suggesting that they had undergone strong phenotypic maturation^45^. MHC-II^+^ neutrophils have recently been linked to immunogenic cell death (ICD), during which they phagocytose dying tumor cells and mediate respiratory-burst-dependent cytotoxicity against residual cells^45^. Interestingly, panobinostat induced the highest cell kill among the seven drugs tested (Fig. 4e,f). Based on our observation of significant enrichment of MHC-II^+^ antigen-presenting neutrophils associated with cell death, we hypothesized that panobinostat-mediated cell death would be immunogenic and the efficacy of this targeted therapy would be enhanced by PD-1 blockade.

Systemic treatment of EMT6 and E0771 model tumors with panobinostat plus anti-PD-1 increased survival duration and reduced tumor growth rate relative to treatment controls or to treatment with panobinostat alone (Figs. 4g and 6c), indicating effective induction of anti-tumor immunity. Consistent with this, systemic treatment with panobinostat significantly increased the proportion of intratumoral CD8^+^ T cells as compared to stromal parenchyma (Extended Data Fig. 5d). However, the treatments did not achieve long-term tumor control (Fig. 4g), and, in vaccination studies^46^, only a subset of mice in both EMT6 and E0771 models rejected the tumor after re-challenge (Fig. 4h). These results suggest that resistance mechanisms exist that might counter the full potential of panobinostat-mediated anti-tumor immunity, and, thus, we explored this treatment condition in more detail.

### Biomarkers of response and mechanisms of resistance associated with early induced anti-tumor immunity in breast cancer

**Fig. 5:**
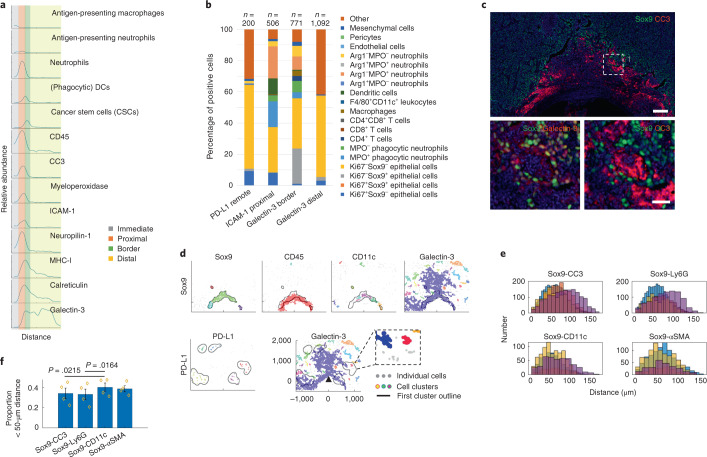
Spatial single-cell analyses of immunogenic cell death biomarkers and associated resistance mechanisms. **a**, Profile plot of the relative abundance of standard cell types and individual biomarkers with distance from the well. Assay zones are color-coded in the legend; profile area is shown in Extended Data Fig. 6a. **b**, Percentages of cells expressing biomarkers of ICD on standard cell types presented in the form of a stack bar graph. The number of cells quantified (*n*) is presented. **c**, A composite image showing mutually exclusive staining of Sox9 and CC3 and co-expression of Sox9 with galectin-3 (bottom left image). Scale bars, 100 μm and 25 μm for top and bottom images, respectively. **d**, Distance-based clustering of depicted marker-positive cells in *x*–*y* coordinates with overlay (black line) with Sox9 (top) and PD-L1 cluster border (bottom), respectively. Individual clusters were identified by a minimum of ten cells within a maximum 50-μm distance for all but the PD-L1 marker, which clustered with a maximum distance set to 150 μm. **e**,**f**, Number of Sox9^+^ pairwise distances with other marker-positive cells presented in the form of a histogram (**e**) and bar graph showing average proportion of Sox9 pairwise distances that were less than 50 µm (**f**). Bars are mean ± s.e.m.; *n* = 4 regions of interest of 175-µm diameter in the border assay zone. Significance was determined by paired two-tailed *t*-test.

**Extended Data Fig. 6:**
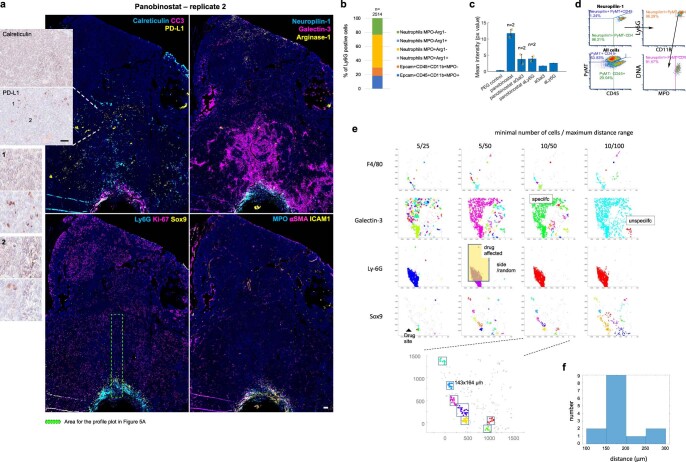
Biomarkers of immunogenic cell death and associated mechanisms of resistance induced by local panobinostat drug stimulus. **a**, Large field of view three-color composite images showing biomarkers assocated with immunogenic cell death induced by panobinostat at three days of exposure. Calreticulin and PD-L1 IHC (red AEC signal) overlayed on hematoxylin nuclei (in blue) are presented in bright field zoomed image on the left. **b**, Expression rate of CD45, MPO and arginase-1 on Ly6G+ cells in the panobinostat assay area to stratify phagocytic, cytotoxic and immune suppressive neutrophils, respectively. Number (*n*) of analyzed cells is presented. **c**, Panobinostat reservoirs were co-loaded with anti-Ly6G (clone 1A8) and galectin-3 (clone M3/38) antibodies at 5:1 to 10:1 ratio and CC3 IHC signal was quantified at the drug releasing site. Bars are mean ± s.e.; *n* = 2 for experimental and 1 for control conditions, respectively. All results were obtained from a single IMD in one tumor which was implanted for two instead of typical three days to account for antibody half-live. MMTV-PyMT mice with late stage spontaneously growing tumors were used. **d**, Image cytometry measuring neuropilin-1 expression on cytotoxic neutrophils. For comparison, population distribution of all cells is presented on the bottom left. **e**, Distance based cluster analysis testing different cluster size strategies to identify treatment specific cluster formation located above the drug-releasing site versus random cluster formation distant from the well. The function implements a user defined cluster sizes set by minimal number of cells / within maximum distance range in µm (for example 5/25, 5/50, 10/50, 10/100; top) to display clusters in randomized color. Clustering strategies using low cell number (for example 5 cells, first two columns) and large distances (for example 100 µm, right column) show clusters forming unspecifically outside the assay area; Clustering strategy using minimal 10 cells within maximum distance range 50 µm (10/50 column) shows cluster formation specifically above the drug site for all presented markers (F4/80, Galectin-3, Ly6G, Sox9). Magnified Sox9 cluster formation; bottom. **f**, Frequency of Sox9 cluster sizes at the panobinostat well. Cluster size 175 µm in diameter, which was the most prominent, was used for downstream analysis of pairwise proximity measurements of Sox9-positive cells with other markers (Fig. 5e, f).

Through literature review, we generated a list of early in situ biomarkers that have been directly or indirectly linked to ICD, increased tumor CD8^+^ T cell infiltrate and/or ICB efficacy. These include intercellular adhesion molecule 1 (ICAM1)^47,48^, myeloperoxidase (MPO)^48^, calreticulin^16,17,49^, MHC-I^50,51^, galectin-3 (refs. ^47,52^), neuropilin-1 (refs. ^53,54^) and PD-L1 (refs. ^8,9^). We validated the presence of these biomarkers at panobinostat reservoirs (Fig. 5a and Extended Data Fig. 6a) and measured their expression and spatial association in relation with the standard stromal cell types in the assay area (Fig. 5b) as well as CSCs (Epcam^−^CD45^+^ PyMT^+^Ki67^−^Sox9^+^) (Fig. 5c–f)—a subset of tumor cells that have self-renewal and tumor-initiating capacity that often exhibit resistance to anti-cancer treatments^5,55,56^.

ICAM1, MPO and neuropilin-1 were localized in the proximal cell death and neutrophil-rich assay region, whereas PD-L1, galectin-3, MHC-I and calreticulin were localized mostly on tumor cells distal from the well, with the latter two decreasing in abundance with increasing distance from the reservoir (Fig. 5a and Extended Data Fig. 6a). Most (65%) of Ly6G^+^ neutrophils were positive for MPO (Extended Data Fig. 6b), consistent with cytotoxic capacity. Positivity for ICAM1 (Fig. 5b) and the mutually exclusive expression of the immune-suppressive molecule arginase-1 in this population (Extended Data Figs. 5b and 6a) indicate that these are anti-tumor (reported also as N1) rather than pro-tumor (N2) neutrophils^57^. Co-treatment with panobinostat and an anti-Ly6G antibody decreased panobinostat-induced cell death, implying that these neutrophils may have a panobinostat-mediated tumor-killing function (Extended Data Fig. 6c). The vast majority (up to 88%) of neuropilin-1^+^ cells proximal to the panobinostat well were cytotoxic neutrophils (Fig. 5a and Extended Data Fig. 6d), suggesting neuropilin-1 as a novel biomarker of anti-tumor neutrophils in breast cancer—a hypothesis that remains to be functionally tested.

Nuclear expression of Sox9 has been associated with stemness in mammary tissue and mammary carcinoma^5,55,56^. We observed CC3 and nuclear Sox9 staining to be mutually exclusive (Fig. 5c and Extended Data Fig. 5b) at the border of cell death/neutrophil-rich region, providing direct in vivo evidence that the CSCs were resistant to the most potent tumor-killing therapy in our screen. In contrast, galectin-3 and Sox9 were co-expressed in many areas of the border region (Fig. 5c,d), with 22% of galectin-3^+^ cells being CSCs (Fig. 5b). This indicates that galectin-3 might be a new biomarker enriching CSCs in breast cancer. Expression and spatial association of galectin-3 with both response (MHC-I and calreticulin) and resistance (PD-L1 and CSC) mechanisms (Fig. 5a–d) suggest pleiotropic involvement of this protein, which implies that targeting galectin-3 during immunogenic cell death should be carefully considered (Extended Data Fig. 6c).

Finally, we assessed the spatial locations of immune cells within the resistant CSC niche. Three spatial analyses, including macroscopic profile plots of relative cell abundance (Fig. 5a), distance-based cluster analyses (Fig. 5d) and pairwise proximity measurements in Sox9 microcultures (Fig. 5e,f and Extended Data Fig. 6e,f), showed that CD11c^+^ DCs were preferentially located in proximity to CSCs, suggesting functional interactions between the two cell types.

### Combination of panobinostat, venetoclax and anti-CD40 immunotherapy maximizes tumor killing and anti-tumor immunity in mammary carcinoma

**Fig. 6:**
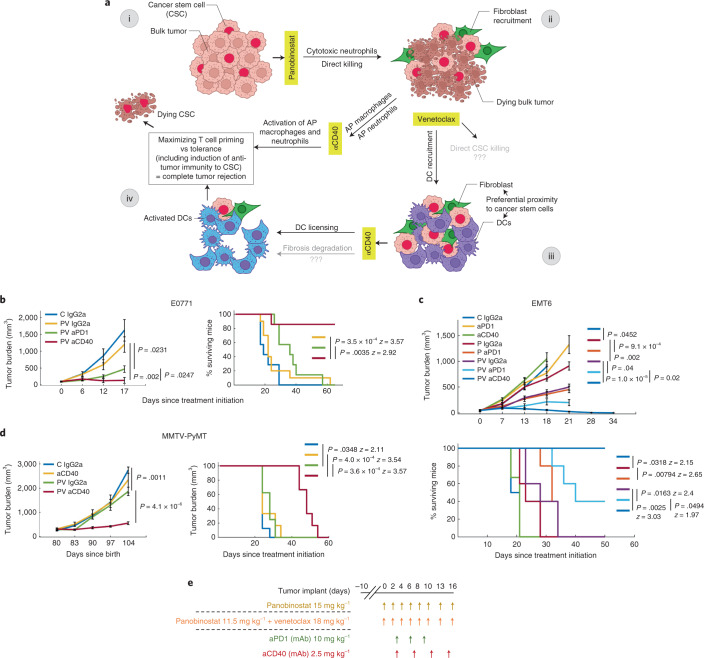
Efficacy of the triple combination of panobinostat, venetoclax and anti-CD40 immunotherapy in mammary carcinoma and rationale for the combination. **a**, Hypothetical model of response for panobinostat/venetoclax/anti-CD40 triple combination treatment efficacy in breast cancer. In brief, the tumor is composed of bulk tumor and CSCs (i). Panobinostat induces immunogenic cell death of the bulk tumor while CSCs remain resistant in the TME (ii). Venetoclax induces recruitment of DCs in proximity to CSCs (iii). We hypothesize that, if CD40 ligation induces licensing of DCs, which captured and processed antigen from neighboring CSCs, the triple combination potentiates CSC-specific anti-tumor immunity, leading to complete tumor rejection (iv). **b**–**d**, Tumor burden measurements (left and top graphs) and survival rate (right and bottom graphs; 100% to 0%) over time in E0771 (**b**); EMT6 (**c**); and orthotopically induced tumor-bearing mice and MMTV-PyMT mice with spontaneously growing tumors (**d**). C, control; P, panobinostat, PV, panobinostat/venetoclax combination. For tumor burden, line graphs are mean ± s.e.m. per timepoint; *n* = 7–10 mice, 6–12 tumors and 6–8 mice per group in **b**, **c** and **d**, respectively. Significance was calculated by unpaired two-tailed *t*-test with equal variance. For survival rate, *P* value was calculated by log-rank (Mantel–Cox) test. **e**, Treatment dose and schedule for **b**,**c**,**d**. Schematics in **a** was partly generated with BioRender. mAb, monoclonal antibody.

The observed spatial association between CSCs and DCs and the observed responses to panobinostat and venetoclax suggested to us a strategy (Fig. 6a) to maximize anti-tumor activity through immune modulation. In this strategy, panobinostat induces immunogenic cell death of bulk tumor while CSCs remain resistant in the TME. Venetoclax induces recruitment of DCs that we have shown to localize to the—now accessible—CSC niche. We hypothesized that CD40 ligation-induced licensing of DCs that had captured and processed antigens from neighboring CSCs would result in activation of CSC-specific anti-tumor immunity, leading to complete tumor clearance. Thus, panobinostat is postulated to induce anti-tumor immunity on the level of bulk tumor, whereas venetoclax/anti-CD40 induces anti-tumor immunity on the level of resistant, tumor-initiating CSCs.

We tested the possibility that the combination of panobinostat/venetoclax (PV) with anti-CD40 immunotherapy would provide maximal therapeutic efficacy in breast cancer. We tested this by systemically treating mice bearing EMT6 and E0771 tumors and compared the responses to those obtained using a PV/anti-PD-1 combination. Treatment with PV/anti-PD-1 significantly reduced the tumor burden as compared to dual PV and panobinostat/anti-PD-1 (Fig. 6b,c) treatments, with survival rates of 40% in mice bearing EMT6 tumors (Fig. 6c). The triple combination of PV/anti-CD40, however, was superior and eliminated measurable tumors in 100% of EMT6 tumors and 85% of E0771 tumors, respectively (Fig. 6b,c). We also assessed the efficacy of PV/anti-CD40 against spontaneous tumors arising in the MMTV-PyMT model and found that this combination inhibited tumor progression and doubled the overall survival (Fig. 6d). Notably, none of the combination treatments in whole animal studies was associated with adverse events, likely because lower systemic concentrations of drugs were used than published previously. We note that two out of eight mice died in the anti-CD40 monotherapy group. Lethal toxicity of anti-CD40 used as a single agent was previously reported due to a shock-like syndrome^58^, and our data also suggest that this immunotherapy is tolerable only with prior administration of anti-cancer agent(s). Although antigen-specific T cell responses remain to be critically evaluated, overall, these results suggest the triple combination of lower-dose panobinostat, venetoclax and anti-CD40 as a highly synergistic therapeutic strategy for long-term breast cancer control.

## Discussion

The MIMA platform described here provides a strategy to design effective combination regimens based on intratumor nanodose exposure to a range of agents (Supplementary Table 4), coupled with highly multiplexed phenotyping and integrated spatial analysis of tumor response to each therapy. The focal drug delivery begins at the time of implantation and can be treated as a spatial and temporal pharmacological perturbation. Because distances from the drug delivery wells reflect recruitment events, analyses of the responses produced by devices left in place provide data about drug-induced changes in cellular densities, molecular phenotypes and possible functional cell interactions. These MIMA-based observations enable development models of drug response that can be used to predict effective TME-modulating combination treatment strategies (Figs. 2f,g, 3f,g and 6a). Many of the drug effects revealed using MIMA are difficult or impossible to study in animal models or humans treated systemically, due to heterogeneous and indeterminate drug distribution that can vary greatly over different regions of a tumor and over time. The TME response patterns obtained from MIMA studies may, in the future, be developed as early in situ biomarkers of therapeutic response, and their further computational processing could provide actionable information to guide the development of effective drug doses and schedules. IMD integration with other analytical approaches, such as metabolic^59^, transcriptomic profiling or electron microscopy, may reveal additional molecular and architectural features of the tumor and TME cell types and states that further inform on drug mechanisms of action.

Although intended as a proof of concept that analyses of local nanodose drug responses can effectively guide systemic treatment strategies, we have already identified specific therapeutic combinations that warrant clinical consideration, including palbociclib/anti-CSF1R, venetoclax/anti-CD40, panobinostat/anti-PD-1 and lower-dose panobinostat/venetoclax/anti-CD40. Clinical work already underway suggests the feasibility of such evaluations. CDK4/6 inhibitors, including palbociclib, are FDA approved and considered as standard of care for patients with metastatic breast cancer^3,60^. A smaller phase 1b study measured the safety and preliminary efficacy of venetoclax in patients with ER^+^BCL2^+^ breast cancer to be similar to other the ‘modern-day’ therapies^61^. Although CDK4/6 inhibitors can induce anti-tumor immunity in breast cancer^62^, in part through epigenetic modulation and antigen presentation^63^, our data did not predict nor show synergy of venetoclax with anti-PD-1, which is in line with previous observations^64^. Instead, we suggest that venetoclax might, through optimal, anti-CD40 immune modulation, target the CSC niche. Future research questions include whether there is a direct effect of this targeted therapy on CSCs (Fig. 6a), what is the role of apoptotic priming^65^ and should venetoclax be used as a common combination partner with other drugs to eliminate the resistant CSCs. Panobinostat-associated human data are limited^66^; however, mouse studies using a more specific, class II HDAC inhibitor, in the same MMTV-PyMT model, showed that infiltration of antigen-presenting macrophages is a mechanism of action in anti-PD-1 therapy response^52^. Our data support this observation, as we saw significant infiltration of the same cell type specifically at the panobinostat condition (Fig. 1g). We extend this knowledge and suggest that, in addition to APC infiltration^52^, induction of immunogenic cell death and proficient antigen presentation machinery in general (both tumor-MHC-I^51^ and MHC-II on different myeloid cells) might be important attributes of effective induction of anti-tumor immunity in breast cancer, and we suggest that epigenetic modulators in general should be considered for ICB synergy in breast cancer. We also showed that probes ICAM1, calreticulin, PD-L1, neuropilin-1, galectin-3 and MPO were spatially associated with immunogenic cell death and, together with the enriched standard cell types, they might serve as an early predictive biomarker of induced anti-tumor immunity in situ. Although ICB immunotherapies are increasingly well-established for breast cancer^51,52,63,64^, anti-CD40 agonists have been evaluated mostly in pancreatic cancer^67^ where their efficacy is party attributed to dense stroma elimination^68^. We observed enrichment of fibroblasts in the CSC niche (Fig. 5e, f). Whether anti-CD40 affects the fibrotic degradation in the niche remains to be determined (Fig. 6a). Nevertheless, considering the strong infiltration of myeloid cells and enrichment of non-immune stroma induced by primary chemotherapies and targeted agents, and the capacity of anti-CD40 to modulate these components to stimulate anti-tumor effects^42–44,68–70^ (Fig. 6a), perhaps anti-CD40 agonists might be the optimal immunotherapy in breast cancer treatment.

We recognize that there are fundamental differences between humans and mice in tumor and immune microenvironment that may influence the performance of drug combinations. Implementation of the MIMA system either directly in humans or perhaps in PDXs or organoid cultures thereof would overcome this limitation. Direct implementation in humans seems best because that would avoid the time, expense and low success rate of establishing human cells in laboratory models. To that end, recent work by Dominas et al.^26^ has demonstrated that the implantable microdevice applications are safe and feasible in patients across multiple cancer indications, including breast, prostate, T cell lymphoma and glioblastoma. Considering the large catalog of FFPE-validated antibodies and well-established mIHC and cycIF workflows for human tissue^27,28,71^, it may become feasible to use the MIMA approach to measure multiple drug responses in individual patients to guide their combination treatment design. Once the assay platform is established, the time from sample collection to data interpretation can take as few as 7–10 days (Methods), which is sufficiently rapid to support clinical decision-making. Notably, the nanoliter amounts of drug delivered by the IMD are sufficiently low that they do not cause systemic toxicity.

We have shown here that drug combinations predicted using MIMA are effective when administered systemically. The predictions take into account the effects of the drugs on both the targeted tumor cells and the associated stromal/immune microenvironment. Notably, our study shows that the effects of drugs nominally developed to target tumor cells also strongly affect the composition and organization of the TME in ways that influence overall tumor response. We also show that including microenvironmental effects in drug combination selection can significantly improve the outcomes of systems treatments. All in all, MIMA represents a new approach to identification of effective combination regimens for individual patients on a personalized basis. Extended use of MIMA will also open new opportunities in in silico modeling to model dynamic drug–tumor–stromal interactions.

## Methods

### Murine models

Mice were purchased from Jackson Laboratory. All animal studies were conducted in accordance with protocols approved by the Institutional Animal Care and Use Committee at Oregon Health & Science University (protocol no. IP00000956). All mice were bred and housed under specific pathogen-free conditions under a standard 12-hour light/dark cycle. C57LB/6, BALB/c and FVB/N mice were purchased from Jackson Laboratory. MMTV-PyMT mice were from Lisa Coussens and purchased from Jackson Laboratory. Virgin female mice 8–24 weeks of age were used for all experiments.

### Cell lines

EMT6 (mouse breast cancer) cells were purchased from the American Type Culture Collection and were maintained in Waymouth’s medium with 10% FBS and 2 mM L-glutamine. E0771 (mouse breast cancer) cells were purchased from CH3 BioSystems and were cultured in RPMI-1640 with 10% FBS and 10 mM HEPES. Both cell lines were pathogen tested and were grown at 5% CO_2_ and 37 °C.

### Experimental design

The objective of the studies in the figures is to show how intact TME responds to local stimulus of drug release and to test whether this response was significantly different from the baseline TME state in tumor regions distant from the drug site. The number of independent biological replicates of each experiment (*n*) performed is given in the figure legend. Spatial systems analyses were designed to quantitatively define directional spatial cell dependencies and cause consequence cell association with distance from the reservoir. These ultimately translated to models of drug response. Within these models, we aimed to identify therapeutic vulnerabilities to predict rational immune or TME-modulating treatment combinations and their optimal schedule and sequencing, which we then validated in traditional whole animal studies.

### Microdevice implantation studies and sample collection

Nanodose drug delivery devices were manufactured and implanted as described previously in ref. ^24^. In brief, cylindrical microdevices 5.5 mm in length and 750 μm in diameter were manufactured from medical-grade Delrin acetyl resin blocks (DuPont) by micromachining (CNC Micro Machining Center) with 18 reservoirs of 200 μm (diameter) × 250 μm (depth) on the outer surface. Reservoirs were packed with drugs mixed with PEG (MW 1450, Polysciences) polymer at the concentrations indicated in Supplementary Table 1. Recommended systemic dose in patients with cancer was derived from the https://rxlist.com web page to June 2017. Systemic doses ranging among 0–1 mg kg^−1^, 1–2 mg kg^−1^, 2–4 mg kg^−1^ and >4 mg kg^−1^ translate to 20%, 25%, 30% and 40% of drug concentration in PEG, respectively, when released from the nanowell. The calibration was determined previously using mass spectrometry measurements^24^. Pure PEG was used in control conditions. Implanting multiple devices per tumor and/or multifocal animal models can increase the throughput up to 50–70 times as compared to conventional systemic treatment studies. Microdevices were implanted for 3 days in MMTV-PyMT with late-stage spontaneously growing tumors in all experiments. Tumor size was between 1.2 cm and 1.5 cm in the longest dimension at the time of implant. Tumors were excised at 3 days after device implantation unless otherwise stated, fixed for 48 hours in 10% formalin or 4% paraformaldehyde and then perfused with paraffin. Specimens were sectioned using a standard microtome, and 5-μm tissue sections were collected from each reservoir. Dry FFPE tissues were baked in a 65 °C oven for 30 minutes. After de-paraffinization with xylene and rehydration in serially graded alcohol to distilled water, slides were subjected to endogenous peroxidase blocking in fresh 3% H_2_O_2_ for 10 minutes at room temperature. Sections were then stained by mIHC and/or cycIF (Extended Data Fig. 1b,c).

### cycIF

Before iterative cycles of (1) staining, (2) whole slide scanning and (3) fluorophore bleaching, the slides were subjected to heat-mediated antigen retrieval by being immersed in citrate buffer (pH 5.5, HK0809K, BioGenex Laboratories, Citra Plus Antigen Retrieval) for 25 minutes and then briefly rinsed in a hot bath and then immersed in Tris/EDTA buffer (pH 9.0, S2368, Dako Target Retrieval Solution) for 15 minutes, all using a Cuisinart Electric Pressure Cooker (CPC-600N1). Protein blocking was performed for 30 minutes at room temperature with 10% normal goat serum (S-1000, Vector Laboratories) and 1% BSA (BP1600-100) in 1×PBS. (1) Slides were incubated with primary antibody (concentrations defined in Supplementary Table 3) for 2 hours at room temperature while being protected from light in a dark humid chamber. All washing steps were performed for 3 × 2–5 min in 1×PBS while agitating. Slides were mounted with SlowFade Gold antifade mountant with DAPI (S36938) using a Corning Cover Glass (2980-245). (2) Images were acquired using Zeiss Axio Scan.Z1 Digital Slide Scanner (Carl Zeiss Microscopy) at ×20 magnification, after which the coverslips were gently removed in 1×PBS while agitating. (3) Fluorophores were chemically inactivated using 3% H_2_O_2_ and 20 mM NaOH in 1×PBS for 30 minutes at room temperature while being continuously illuminated. The fluorophore inactivation was repeated twice with a short, 10-minute, 1×PBS wash in between. Efficacy of bleaching was imaged before antibody incubation (baseline autofluorescence) and every third to fourth cycle on average. After protein blocking, samples were subjected to the next round of staining. Single-cell feature extraction was not applied to evaluate sections stained by cycIF.

### mIHC

Before iterative cycles of (1) staining, (2) whole slide scanning and (3) heat and chemical stripping of antibodies and chromogen, the slides were subjected to staining with F4/80 and CSF1R antibodies (cycle zero, no antigen retrieval; Supplementary Table 2) and hematoxylin staining (S3301, Dako) for 1–5 minutes, followed by whole slide scanning. Slides were then subjected to the first heat-mediated antigen retrieval in 1× pH 5.5–6 citrate buffer (BioGenex Laboratories, HK0809K) for 90 seconds in a low-power microwave and 16 minutes in a steamer, followed by protein blocking with 10% normal goat serum (S-1000, Vector Laboratories) and 1% BSA (BP1600-100) in 1×PBS for 30 minutes at room temperature. (1) Slides were incubated with primary antibodies (concentrations defined in Supplementary Table 2) for 1 hour at room temperature or 16–17 hours at 4 °C while being protected from light in a dark humid chamber. Signal was visualized with either anti-rabbit or anti-rat Histofine Simple Stain MAX PO horseradish peroxidase (HRP)-conjugated polymer (Nichirei Biosciences), followed by peroxidase detection with 3-amino-9-ethylcarbazole (AEC). Two or three drops of HRP polymer were used for up to nickel-size or whole slide tissue sample, respectively. Timing of AEC development was determined by visual inspection of positive control tissue (Extended Data Fig. 1d–f) for each antibody. All washing steps were performed for 3 × 5–10 minutes in 1×PBS while agitating. Slides were mounted with a filtered 1×PBS with 0.075% Tween 20 (BP337100) using a Signature Series Cover Glass (Thermo Fisher Scientific, 12460S). (2) Images were acquired using the Aperio ImageScope AT (Leica Biosystems) at ×20 magnification, after which the coverslips were gently removed in 1×PBS while agitating. (3) Within one cycle, removal of AEC and HRP inactivation was accomplished by incubating the slides in 0.6% fresh H_2_O_2_ in methanol for 15 minutes. AEC removal and stripping of antibodies was accomplished by ethanol gradient incubation and heat-mediated antigen retrieval such as described above between cycles. After washing and protein blocking, samples were subjected to the next round of staining.

The readout antibody panel was carefully designed so that it broadly captures all major TME subtypes and allows to find synergy with the most established and/or emerging immunotherapies (Supplementary Table 4). Based on this, we defined a minimal essential set of 13 markers that classifies distinct myeloid and lymphoid lineages as well as components of non-immune stroma (for non-immune TME modulation). Staining the baseline discovery set of 13 markers can be completed in 4–7 days considering that 3–4 markers and two markers are currently detected in one cycle (1 day) in the mIHC and cycIF procedures, respectively. Before that, an additional 3 days are required for sample fixation, paraffin embedding and FFPE block cutting, resulting in total turnaround time of 7–10 days from sample collection to data acquisition and interpretation. However, the method is flexible such that markers can be subtracted or added to allow for deeper cell characterization of identified phenotypes based on investigator interest. We also envision that, by accommodating an increased number of markers per cycle (for example, by using spectral deconvolution techniques), we can further reduce the turnaround times.

The cost of the MIMA workflow has two major components: one, the cost of the drug-loaded microdevices, which is ~$600–800 per device for a typical study, depending on the number and cost of individual drugs loaded into the device reservoirs; and two, the cost of the cycIF/mIHC, which is ~$50 per slide per cycle with basic (single-stain) immunohistochemistry infrastructure in place. It should be noted, however, that up to six tumor/device specimens are embedded in a single paraffin block so as to reduce the total number of slides required.

### Image processing and feature extraction of mIHC images

The iteratively digitized images were co-registered using MATLAB (MathWorks, version 2019b) using the detectSURFFeatures algorithm from the Computer Vision Toolbox. The imperfectly registered images were additionally processed using the Linear Stack Alignment with SIFT plugin (Fiji) so that cell features overlap down to a single-pixel level. Hematoxylin-stained images were color deconvoluted for single-cell nuclear segmentation to generate a binary mask using the watershed function and standard image processing steps (noise removal, erosion and dilation; Fiji)^72^. AEC chromogenic signal was extracted using the NIH plugin RGB_to_CMYK to separate AEC signal into the yellow channel for improved sensitivity of immunohistochemistry evaluation^73,74^. Grayscale images of all proteins and the binary mask were imported to CellProfiler (version 3.1.8, Broad Institute)^75^ to quantify single-cell signal mean intensity as defined by mask, which was scaled to a range of 0–1. The IdentifyPrimaryObjects module was used to identify nuclei from mask; the MeasureObjectIntensity module measured mean intensity for each object for each protein. The mean signal intensity per cell output was imported to FCS Express 6 and 7 Image Cytometry Software (De Novo Software) to perform multidimensionality reduction to classify ‘standard cell types’. Gating strategies and hierarchical cell classification are presented in Fig. 1e and Extended Data Fig. 2e. Polygonal gates moving around the central vertex without changing the polygon shapes were used to obtain quantitatively reproducible multiplex data, batch to batch, independent of the condition measured. Positive control tissues were used to help define the single-parameter threshold for positivity by manual gating. A total of 3,000–5,000 cells were analyzed for feature extraction in the assay area located above the drug-releasing site with ±300 total cells for paired, experimental versus control, region. Minimum population proportion within 5% margin of error and 95% confidence level was set to 0.75% (represents 12 cells) to discriminate noise from specific cell enrichment induced by, for example, increased protein expression or cell recruitment into the assay region. Experimental condition of the assay area was compared to random control intratumoral region located perpendicular and/or far from the drug-releasing reservoir. To obtain greater control over confounding variables, paired sample one-tailed *t*-tests were used to determine enrichment of induced TME states. Percentage of positivity and significance were presented in form of a heat map or bar graphs. Quality of the single-cell data was ensured by excluding deformed (folded), lost or unevenly stained tissue (border effects). The assay area was determined by the first 3,000–5,000 cells above the well excluding these deformed regions. Single-cell data from FCS Express were extracted in a data grid to MATLAB for downstream spatial systems analyses. In computed images, neutrophils are presented independent of the Epcam^+/−^ status.

### Spatial systems analyses

The distance-based cluster function finds clusters in a set of spatial points expressed in *x*–*y* space (adapted and modified from Yann Marcon; MATLAB October 2019). The clustering is based on Euclidean distance between the points (cells). The function does not require the number of clusters to be known beforehand. Each cell clusters with the closest neighboring cell if the distance between the two cells is shorter than the defined threshold. The minimal number of cells per cluster is defined by the user. The function outputs non-clustering cells in gray color, and each cluster meeting the defined parameters (minimal number of cells within maximum distance range) is presented in randomized colors. Clusters within the maximum defined distance merge and share one color. Number of clusters and total coverage in the assay area were calculated using distinct cluster sizes (defined by minimal number of cells within maximum distance range) for control PEG and palbociclib, which identified that cells cluster in response to treatment if a minimum of ten cells are present within a maximum distance range of 30–75 μm (systematic comparison not shown in this study). Cluster parametrization using as few as five cells and distances as large as 100 μm resulted in treatment non-specific cluster formation in PEG negative control. Treatment-specific cluster formation with cluster definition of a minimum of ten cells within 50-μm distance was generalizable to all marker and standard cell types, which was confirmed in panobinostat condition by comparing assay area and distal region side by side in one field of view (Extended Data Fig. 6e). This treatment-specific cluster parametrization was applied in downstream analytics to identify hotspots/zones of interest (for example, proximal, border, distal, network adjacent, CD11c^+^ DC clusters) in an objective, biology-driven manner.

For the relative abundance profile plot, marker-positive cells and the standard cell types were extracted to *x*–*y* coordinate space; signal was blurred using Gaussian blur filter; and relative abundance of positive cells was displayed with distance from the well in a profile plot as outlines in corresponding Extended Data figures. A moving average filter with 50-μm and 100-μm window size (movmean function, MATLAB) was additionally applied to smoothen the feature signal for palbociclib and panobinostat condition, respectively. Signal in the profile plots was not scaled.

Inside the hotspot, spatial (geographical) interactions between marker-positive cells were determined by proximity measurements in local microculture by using the pdist2 function in MATLAB (version 2019b), which returns the distance of each pair of observations (positive cells) in *x* and *y* using metric specified by Euclidean distance. Random circular regions of 175-μm diameter (defined by Extended Data Fig. 6f) were selected in the border, CSC-rich zone of the panobinostat assay area, and Euclidean distance was measured between Sox9^+^ and other marker-positive cells. The number of distances was presented in the form of a histogram. To quantify spatially interrelated phenomenon, proportions of distances lower than 50 μm (as defined by distance-based cluster analyses) were compared between different cell pairs (for example, Sox9^+^Ly6G^+^ versus Sox9^+^CD11c^+^).

Extended hierarchical cell classification was applied to characterize the significantly enriched cell phenotypes forming zones of interest that were outside the standard cell type classification (for example, less-differentiated macrophages or phagocytic DCs). Probe combination and number of cells analyzed within number of clusters are defined in the figures and figure legends.

Two-dimensional composite images were presented by using Fiji^72^.

The spatial systems analyses were used to identify drug models of response (presented as line diagrams), and the identified therapeutic vulnerabilities were tested in whole animal studies.

### Whole animal treatment studies

Although the high-throughput IMD experiments were performed in the MMTV-PyMT model^30,31,76,77^ with spontaneously growing tumors, the whole animal validation studies of predicted immune-modulating combinations were performed using transplantable breast cancer cell lines in syngeneic mice to avoid extensive breeding and colony maintenance necessary to test synergy of multiple predicted combinations. E0771 and EMT6 models, which are typically used in breast cancer research involving immunotherapy testing^32,78,79^, were selected randomly for validation of different combinations. The combination of panobinostat and anti-PD-1 was tested in both transplantable models. The most potent triple combination of panobinostat, venetoclax and anti-CD40 was additionally tested in the MMTV-PyMT model with spontaneously growing tumors.

The MMTV-PyMT model has a 100% penetrance and shows good consistency in latency times and similar tumor characteristics^76^. The model was developed in 1992 in the Muller laboratory^30^, and, despite the PyMT not being a human oncogene, it mimics the signaling of RTKs, which are often activated in human malignancies, including breast cancer. PyMT expression under the MMTV promoter results in rapid transformation and generation of multifocal tumors that metastasize to lungs. Tumors arising in luminal cells progress through distinct histological stages that mimic human ductal breast cancer progression (hyperplasia, adenoma, MIN and early and late carcinoma)^31^. Loss of ER and PR expression is observed as the disease progresses^31^. By gene expression profiling, this model clusters with luminal B subtype^32,80,81^.

Transcriptionally, the orthotopic syngeneic models fall into luminal A (EMT6) and luminal B (E0771) intrinsic subtype despite being aggressive with poorly differentiated or spindle-shaped histopathology. Both models showed transcriptomic characteristics of ‘claudin-low’ human subtype with a high score for EMT, low differentiation and low proliferation^82^.

MMTV-PyMT transgenic mice that were 80 days of age were randomized and included in the study when their total tumor burden was 150–550 mm^3^ (treatment initiation). For the orthotopically induced tumor models of mammary carcinoma, EMT6 (0.5 × 10^6^ in 1×PBS per site) and E0771 (0.5 × 10^6^ in Corning matrigel per site) cells were injected into the #4 mammary fat pad of female virgin BALB/c and C57LB/6, respectively. One tumor was induced in the E0771 and two tumors were induced in the EMT6 model. Caliper measurements were used to calculate the tumor volumes using the formula length × width^2^ / 2. Treatments were initiated when total tumor burden was 60–150 mm^3^. For all models, the endpoint was determined by tumor volume above 2,000 mm^3^ in two consecutive measurements or one measurement above 2,200 mm^3^. Treatments were administered by intraperitoneal injection. Dose, schedule and duration are indicated in the respective figures and figure legends. We note that the doses for panobinostat and venetoclax were decreased from 15 mg kg^−1^ to 11.5 mg kg^−1^ and from 22 mg kg^−1^ to 18 mg kg^−1^, respectively, when the two drugs were combined (Fig. 6e). Treatment schedule was estimated depending on the location of the targetable cell phenotype in proximity to the well or more distal from the drug source. For example, cells in the immediate proximity of the drug well at 3 days of exposure were likely recruited first to the drug assay area; thus, early targeting (pre-treatment) of these cells is preferred. Inversely, cells located in distal regions should be targeted by post-treatment approach. Diluent and IgG2a isotype control (Bio X Cell) concentrations were equivalent to the highest dose of the respective drug used in each experiment. Mice that survived the first treatment cycle were allotted an 8-day break before the start of one additional treatment cycle with the same administration of drug doses and duration.

The mice were monitored daily to determine any possible effects on the general condition of the animals using parameters as established by Morton and Griffiths (1985). The guidelines for pain, discomfort and distress recognition were used to evaluate weight loss, appearance, spontaneous behavior, behavior in response to manipulation and vital signs. Specifically, general appearance (dehydration, missing anatomy, abnormal posture, swelling, tissue masses and prolapse), skin and fur appearance (discoloration, urine stain, pallor, redness, cyanosis, icterus, wound, sore, abscess, ulcer, alopecia and ruffled fur), eyes (exophthalmos, microphthalmia, ptosis, reddened eye, lacrimation, discharge and opacity), feces (discoloration, blood in the feces and softness/diarrhea) and locomotor (hyperactivity, coma, ataxia and circling) were monitored to determine loss of body condition (BC) score, namely: BC 1 (emaciated) score was applied when skeletal structure was extremely prominent with little or no flesh/muscle mass and vertebrae was distinctly segmented; BC 2 (under-conditioned) score was applied when segmentation of vertebrate column was evident, dorsal pelvic bones were readily palpable and muscle mass was reduced; and BC 3 (well-conditioned) was applied when vertebrae and dorsal pelvis were not prominent/visible and were palpable with slight pressure. Loss of BC was also considered when anorexia (lack or loss of appetite) or failure to drink; debilitating diarrhea and dehydration/reduced skin turgor; edema, sizable abdominal enlargement or ascites, progressive dermatitis, rough hair coat/unkempt appearance, hunched posture, lethargy, loss of righting reflex, neurological signs or bleeding from any orifice appeared in treated mice. Most treated groups were well-conditioned (BC score 3); less than 20% of mice in each group experienced mild diarrhea for up to 2 days once during the course of treatment (typically after the first or second therapy administration). Mice receiving palbociclib monotherapy were under-conditioned (BC score 2) starting from day three until the end of the treatment. Two out of eight mice in the MMTV-PyMT model died within 1–3 days after the first injection of αCD40 immunotherapy when administered as a single agent. Lethal toxicity of anti-CD40 used as a single agent was previously reported due to a shock-like syndrome^58^, and our data also strongly suggest that this immunotherapy is tolerable only with prior administration of anti-cancer agent(s). Surviving mice receiving venetoclax/anti-CD40 combination experienced fur graying to different degrees starting approximately 4 weeks after treatment. No signs of pain, discomfort or distress were observed in the surviving mice. Neither emaciated (BC score 1), over-conditioned (BC score 4) nor obese (BC score 5) were observed in our studies.

To show CD8^+^ T cell infiltration inside the tumor bed, ErbB2ΔEx16 mice^83^ with spontaneously growing late-stage tumors were intraperitoneally injected with panobinostat (15 mg/mg) on day zero, day two and day four. Tumors were extracted at day seven, were FFPE processed and were stained for CD8 to compare the rate of intratumoral CD8^+^ T cells in panobinostat-treated versus control (diluent)-treated mice.

### Vaccination study

EMT6 and E0771 cells in tissue culture were treated with a soluble drug panobinostat at 5 μM concentration when they would reach 60–70% confluency. After 2 days, the cells were harvested and were injected subcutaneously (total 2–3 × 10^6^ cells) into the lower left flank of BALB/c and C57Bl6 mice, respectively. Cells freeze–thawed three times served as negative control for non-immunogenic form of cell death. After 7–8 days, the mice were re-inoculated by injecting living cells orthotopically into one #4 mammary fat pad (total 0.5 × 10^6^ cells), and tumor appearance was monitored by minimal tumor size approximately 5 mm and 3.5 mm in the longest dimension for E0771 and EMT6 models, respectively (palpable tumors). We note that the E0771 tumors after re-challenge appeared at the primary subcutaneous site, and no tumors were developed in the orthotopic site.

### Statistics and reproducibility

All data are combined from 2–3 independent experiments, unless specifically noted. To accomplish randomization for systemic mouse experiments, animals were sorted by a blinded investigator, and then groups were assigned. Each group was checked post hoc to verify no statistical significance in average starting tumor size. There was no sample size estimation in standard drug treatment experiments. Data are shown as mean ± s.e.m., unless otherwise noted. For tumor growth rate, significance was calculated by unpaired two-tailed *t*-test with equal variance. For survival and tumor-free analyses, Kaplan–Meier curves were generated to demonstrate time to event, and the log-rank (Mantel–Cox) test was used to evaluate statistical significance. For representative micrographs, each experiment was repeated at least three times with similar results, unless stated otherwise.

### Reporting summary

Further information on research design is available in the Nature Research Reporting Summary linked to this article.

## Online content

Any methods, additional references, Nature Research reporting summaries, source data, extended data, supplementary information, acknowledgements, peer review information; details of author contributions and competing interests; and statements of data and code availability are available at 10.1038/s41587-022-01379-y.

## Supplementary information

Supplementary Information Supplementary Table 1 | Drug list and drug concentration calibration used in the MIMA system. Supplementary Table 2 | Antibody order, catalog and concentration used in the mouse mIHC. Supplementary Table 3 | Antibody order, catalog and concentration used in the mouse cycIF. Supplementary Table 4 | Rationale to select effective TME-modulating combination treatments based on the intratumoral drug–response signature

Reporting Summary

## Data Availability

Source raw registered images for feature extraction in the drug assay region will be provided as a collection of images per condition (Figs. 2–4) at the figshare.com repository: 10.6084/m9.figshare.19719421, 10.6084/m9.figshare.19719499 and 10.6084/m9.figshare.19719514. All other data that support the findings of this study are available in the article, in its Supplementary Information or from the corresponding authors upon reasonable request.

## Code availability

The image analysis pipeline using publicly available functions is detailed in the Methods section, in the article and in its Supplementary Information. The associated scripts are deposited at https://github.com/ztatarova/MIMA-study.
