# Supplementary material for: A multiplex implantable microdevice assay identifies synergistic combinations of cancer immunotherapies and conventional drugs
Source: Nat Biotechnol. 2022 Jul 4;40(12):1823–33. doi: 10.1038/s41587-022-01379-y (PMC9750874; doi:10.1038/s41587-022-01379-y)
Supplement: Supplementary file 1 — Supplementary Table 1 | Drug list and drug concentration calibration used in the MIMA system. Supplementary Table 2 | Antibody order, catalog and concentration used in the mouse mIHC. Supplementary Table 3 | Antibody order, catalog and concentration used in the mouse cycIF. Supplementary Table 4 | Rationale to select effective TME-modulating combination treatments based on the intratumoral drug–response signature [file 41587_2022_1379_MOESM1_ESM.pdf]

nature portfolio

https://doi.org/10.1038/s41587-022-01379-y

Supplementary information

A multiplex implantable microdevice assay identifies synergistic combinations of cancer immunotherapies and conventional drugs

In the format provided by the authors and unedited

Supplementary Table 1 | Drug list and drug concentration calibration used in the MIMA system

Drug list	Target	#Systemic dosing	Systemic dosing (mg/kg)	*Concentration in the nanowell
Doxorubicin	DNA breaks	105mg/3weeks	1.05-2.1mg/kg	25%
Paclitaxel	Mitotic inhibition	130mg/3weeks	1.3-2.6mg/kg	25%
Olaparib	PARP	800mg/day oral	8-16mg/kg	40%
Palbociclib	CDK4/6	125mg/daily	1.25-2.5mg/kg	25%
Lenvatinib	VEGFR1/2/3	24mg/daily	0.24-0.48mg/kg	20%
Panobinostat	HDAC	20mg/3x a week	0.2-0.4mg/kg	20%
Venetoclax	BCL2	20-400mg/kg	0.2-8mg/kg	30%

*# Recommended systemic dose was derived from the https://rxlist.com web page to April 2017. * Systemic doses ranging between 0-1mg/kg, 1-2mg/kg, 2-4mg/kg, >4mg/kg translate to 20%, 25%, 30% and 40% of drug concentration in PEG, respectively, when released from the nanowell. The calibration was determined using mass spectrometry measurements (Jonas et al., 2015).*

Supplementary Table 2 | Antibody order, catalog and concentration used in the mouse multiplex immunohistochemistry

Cycle	Anti-rabbit	Anti-rat
0	CSF-1R	F4/80
	Santa Cruz, 1:250	BioRAD, 1:200
	sc-692	MCA497
1	Sox9	MHC-II
	CST, 1:100	Biolegend, 1:150
	D8G8H	M5/114.15.2
1	Hematoxylin	
	Dako, 50 seconds	
	S330-130-2	
2	CD4	CD8
	CST, 1:150	eBiosciences
	D7D2Z	4SM15
3	CD3	CD45
	Thermo, 1:300	BD Pharmingen, 1:60
	SP7	30-F11
4	CD11c	CD45R
	CST, 1:150	BD Biosciences, 1:200
	D1V9Y	RA3-6B2
5	aSMA	CD31
	Abcam, 1:250	Dianova, 1:40
	Ab5694	SZ31
6	MHC-I	PyMT
	Biorbyt, 1:120	Novus, 1:125
	Orb135651	NB100-2749
7	MPO	Ly6G
	Thermo, 1:200	BD Pharmigen, 1:150
	Rb-373-A	1A8
8	ICAM-1	Galectin-3
	Sino, 1:200	Biolegend, 1:400
	50440-R280	M3/38
9	Ki67	
	CST, 1:200	
	D3B5	
10	CD40	
	CST, 1:200	
	E2Z7J	
11	GzmB	FoxP3
	CST, 1:100	Invitrogen, 1:50
	D6E9W	14-5773-82
12	Epcam	
	CST, 1:120	
	E6V8Y	
13	CC3	
	CST, 1:200	
	Asp175	
14	PDL1	
	CST, 1:200	
	D5V3B	
15	Arg-1	
	CST, 1:200	
	D4E3M	
16	Calreticulin	
	Abcam, 1:550	
	Ab92516	
17	CD11b	
	Abcam, 1:5000	
	EPR1334	
18	NRP1	
	Abcam, 1:3000	
	Ab81321	

Sino, SinoBiological; CST, Cell Signaling Technology

Supplementary Table 3 | Antibody order, catalog and concentration used in the mouse cyclic immunofluorescence

Cycle	Fluorophore conjugated to primary antibody			
	488	555	647	750
1		PD-L1	Sox9	MHC-II
		CST, 1:120	CST, 1:150	Biolegend, 1:150
		D5V3B	D8G8H	M5/114.15.2
2		CD4	CD11c	CD103
		CST, 1:100	CST, 1:120	Biolegend, 1:150
		D7D2Z	D1V9Y	2E7
3		Epcam	Arginase-1	CD8
		Sino, 1:120	CST, 1:200	eBiosciences, 1:120
		50591-R002	D4E3M	4SM15
4	αSMA	CD3	CC3	CD31
	CST, 1:150	CST, 1:100	CST, 1:120	Abcam, 1:150
	D4K9N	D4V8L	ASP175	EPR17260
5		CD45	Keratin-14	ICAM-1
		CST, 1:100	Abcam, 1:200	Sino, 1:120
		D3F8Q	EPR17350	50440-R280
6		CD11b	Ki-67	Foxp3
		Abcam, 1:100	CST, 1:120	Novus, 1:150
		EPR1344	D3B5	NB100-39002
7		Desmin	Galectin-3	Myeloperoxidase
		Abcam, 1:150	Biolegend, 1:150	R&D, 1:200
		[Y66] ab32362	125408	AF3667
8	Collagen-VI	F4/80	NF-kB	Elastin
	Abcam, 1:150	CST, 1:120	CST, 1:100	SAB, 1:150
	EPR17072	D2S9R	D14E12	C45078-AF750
9		CSF-1R	MMP-2	Collagen-IV
		Sino, 1:100	Abcam, 1:200	MDBiosciences, 1:120
		50059-T24	EPR1184	203003
10		Granzyme B	Vimentin	ESR1
		CST, 1:100	CST, 1:200	Sino, 1:120
		D6E9W	D21H3	106132-T08
11		E-cadherin	Nox-4	Fibronectin
		Sino, 1:120	Abcam, 1:100	Abcam, 1:1000
		50671-RP02	UOTR1B492	[F14] ab45688
12		HSP-47	CD40	Axl
		Abcam, 1:100	CST, 1:80	R&D, 1:100
		EPR4217	E2Z7J	AF854

Sino, SinoBiological; CST, Cell Signaling Technology

Supplementary Table 4 | Rationale to select effective TME modulating combination treatments based on the intratumoral drug-response signature

Phenotype signature	Rational TME modulating combination	Example from the current study	Purpose
protumor macrophages and/or CSF1R+ cells	CSF1/CSF1R axis targeting agents	Palbociclib + anti-CSF1R	To reduce neovascularization and pro-proliferative TME
All myeloid cells but protumor macrophages; DCs preferred	anti-CD40 agonist antibody	Panobinostat, Venetoclax or both + anti-CD40	To shift balance from immune tolerance to antitumor priming
Immunogenic cell death, MHCII+ neutrophils and ICAM-1+ cells	Anti-PD-1/PD-L1 antibodies	Panobinostat + anti-PD-1	To harvest increased tumor immunogenicity
CD31 and/or aSMA associated cells	VEGFR inhibitors, angiotensin blockers and anti-fibrotics	All targeted therapies and doxorubicin + e.g. Losartan (not tested)	To limit non-immune stromal barriers of drug/cell access

[a]*Protumorigenic macrophages and other CSF1R+ cells can be targeted by immunotherapies affecting CSF1/CSF1R axis.* [b]*All myeloid cells but protumor (M2) macrophages (Verreck et al., 2006) can be modulated by anti-CD40 immunotherapy to potentiate anti-tumor immunity, with dendritic cells being most potent to activate naïve T cells to become effectors (Mempel et al., 2013).* [c]*Drugs inducing immunogenic cell death associated with proficient antigen presentation machinery, ICAM-1, galectin-3 and neuropilin-1signature will synergize with immune checkpoint blocking immunotherapies.* [d]*All of the evaluated targeted agents as well as doxorubicin induced significant changes in non-immune stromal compartment suggesting normalization of vasculature, pericytes and dense stroma inhibition might potentiate the efficacy of chemotherapies, immunotherapies and/or targeted anti-cancer agents in breast cancer.*
