# Supplementary material for: A multiplex implantable microdevice assay identifies synergistic combinations of cancer immunotherapies and conventional drugs
Source: Nat Biotechnol. 2022 Jul 4;40(12):1823–33. doi: 10.1038/s41587-022-01379-y (PMC9750874; doi:10.1038/s41587-022-01379-y)
Supplement: Supplementary file 2 — Reporting Summary [file 41587_2022_1379_MOESM2_ESM.pdf]

nature research

Corresponding author(s): Oliver Jonas, Joe W Gray

Last updated by author(s): 2022-05-04

Reporting Summary

Nature Research wishes to improve the reproducibility of the work that we publish. This form provides structure for consistency and transparency in reporting. For further information on Nature Research policies, see our Editorial Policies and the Editorial Policy Checklist.

Please do not complete any field with "not applicable" or n/a. Refer to the help text for what text to use if an item is not relevant to your study.
For final submission: please carefully check your responses for accuracy; you will not be able to make changes later.

Statistics

For all statistical analyses, confirm that the following items are present in the figure legend, table legend, main text, or Methods section.

n/a	Confirmed	
☐	☒	The exact sample size (*n*) for each experimental group/condition, given as a discrete number and unit of measurement
☐	☒	A statement on whether measurements were taken from distinct samples or whether the same sample was measured repeatedly
☐	☒	The statistical test(s) used AND whether they are one- or two-sided<br>*Only common tests should be described solely by name; describe more complex techniques in the Methods section.*
☒	☐	A description of all covariates tested
☐	☒	A description of any assumptions or corrections, such as tests of normality and adjustment for multiple comparisons
☐	☒	A full description of the statistical parameters including central tendency (e.g. means) or other basic estimates (e.g. regression coefficient) AND variation (e.g. standard deviation) or associated estimates of uncertainty (e.g. confidence intervals)
☐	☒	For null hypothesis testing, the test statistic (e.g. *F*, *t*, *r*) with confidence intervals, effect sizes, degrees of freedom and *P* value noted<br>*Give P values as exact values whenever suitable.*
☒	☐	For Bayesian analysis, information on the choice of priors and Markov chain Monte Carlo settings
☐	☒	For hierarchical and complex designs, identification of the appropriate level for tests and full reporting of outcomes
☒	☐	Estimates of effect sizes (e.g. Cohen's *d*, Pearson's *r*), indicating how they were calculated

Our web collection on statistics for biologists contains articles on many of the points above.

Software and code

Policy information about availability of computer code

Data collection	Zeiss Axio Scan.Z1 Digital Slide Scanner (Carl Zeiss Microscopy), Aperio ImageScope AT (Leica Biosystems), Matlab (The MathWorks, Inc., Natic, MA, version 2019b), ImageJ/Fiji (Schneider et al., 2012; version 2.0.0-rc-69/1.52p), CellProfiler (version 3.1.8, Broad Institute), FCS Express 6 and 7 Image Cytometry Software (DeNovo Software), Microsoft Excel (version 16.54)
Data analysis	The iteratively digitized images were co-registered using Matlab (The MathWorks, Inc., Natic, MA, version 2019b) utilizing the detectSURFFeatures algorithm. The imperfectly registered images were additionally processed using the Linear Stack Alignment with SIFT plugin (Fiji) so that cell features overlap down to a single pixel level. Images were color deconvoluted for single cell nuclear segmentation using watershed function and the AEC chromogenic signal was extracted using the NIH plugin RGB_to_CMYK. Gray scale images were imported to CellProfiler (version 3.1.8, Broad Institute) to quantify single cell signal mean intensity as defined by mask which was scaled to a range 0-1. IdentifyPrimaryObjects module was used to identify nuclei from mask; MeasureObjectIntensity module measured mean intensity for each object for each protein. The mean signal intensity per cell output was imported to FCS Express 6 and 7 Image Cytometry Software (DeNovo Software) to perform multidimensionality reduction to classify "cell standards".<br>Distance based cluster function was adapted and modified from Yann Marcon; Matlab October 2019. For the relative abundance profile plots movmean function (The MathWorks, Inc., Natic, MA, version 2019b) was used to smoothen the feature signal. The spatial (geographical) interactions between marker positive cells were determined by proximity measurements using the pdist2 function (MathWorks, Inc., Natic, MA, version 2019b). No custom software or code was used for data analysis in this study.<br>Caliper measurements were used to calculate the tumor volumes using formula length x width2 / 2 in Microsoft Excel (version 16.54).

For manuscripts utilizing custom algorithms or software that are central to the research but not yet described in published literature, software must be made available to editors and reviewers. We strongly encourage code deposition in a community repository (e.g. GitHub). See the Nature Research guidelines for submitting code & software for further information.

Data

Policy information about availability of data

All manuscripts must include a data availability statement. This statement should provide the following information, where applicable:

- Accession codes, unique identifiers, or web links for publicly available datasets
- A list of figures that have associated raw data
- A description of any restrictions on data availability

The source raw registered images for feature extraction in the drug assay region will be provided as a collection of images per condition (Figs. 2, 3 and 4) at the Mendeley Data repository https://data.mendeley.com/v1/datasets/t3j2j4s6j6/draft with published manuscript (link to be updated). All the other data that support the findings of this study are available within the article, its Supplementary Information, or from the corresponding authors up on reasonable request.

Field-specific reporting

Please select the one below that is the best fit for your research. If you are not sure, read the appropriate sections before making your selection.

☒ Life sciences ☐ Behavioural & social sciences ☐ Ecological, evolutionary & environmental sciences

For a reference copy of the document with all sections, see nature.com/documents/nr-reporting-summary-flat.pdf

Life sciences study design

All studies must disclose on these points even when the disclosure is negative.

Sample size	Total of 3000-5000 cells were analyzed for feature extraction in the assay area located above the drug releasing site with +/- 300 total cells for paired, experimental vs control, region. Minimum population proportion within 5% margin of error and 95% confidence level was set to 0.75% (represents 12 cells) to discriminate noise from specific cell enrichment induced by e.g. increased protein expression or cell recruitment into the assay region. No statistical methods were used to predetermine sample sizes for the whole animal in vivo experiments but the sample sizes are similar to those reported in previous publications.
Data exclusions	Quality of the single cell data was ensured by excluding deformed (folded), lost or unevenly stained FFPE tissue (border effects). Animals were excluded only if they died or had to be killed according to our IACUC protocol. This applied specifically to two
Replication	The number of independent biological replicates of each experiment (n) performed are given in the figure legends. All whole animal data are combined from two to three independent experiments, unless specifically noted.
Randomization	To accomplish randomization for systemic mouse experiments, animals were sorted by a blinded investigator and then groups were assigned. Each group was checked post-hoc to verify no statistical significance in average starting tumor size.
Blinding	Animals were sorted by a blinded investigator.

Behavioural & social sciences study design

All studies must disclose on these points even when the disclosure is negative.

Study description	*Briefly describe the study type including whether data are quantitative, qualitative, or mixed-methods (e.g. qualitative cross-sectional, quantitative experimental, mixed-methods case study).*
Research sample	*State the research sample (e.g. Harvard university undergraduates, villagers in rural India) and provide relevant demographic information (e.g. age, sex) and indicate whether the sample is representative. Provide a rationale for the study sample chosen. For studies involving existing datasets, please describe the dataset and source.*
Sampling strategy	*Describe the sampling procedure (e.g. random, snowball, stratified, convenience). Describe the statistical methods that were used to predetermine sample size OR if no sample-size calculation was performed, describe how sample sizes were chosen and provide a rationale for why these sample sizes are sufficient. For qualitative data, please indicate whether data saturation was considered, and what criteria were used to decide that no further sampling was needed.*
Data collection	*Provide details about the data collection procedure, including the instruments or devices used to record the data (e.g. pen and paper, computer, eye tracker, video or audio equipment) whether anyone was present besides the participant(s) and the researcher, and whether the researcher was blind to experimental condition and/or the study hypothesis during data collection.*
Timing	*Indicate the start and stop dates of data collection. If there is a gap between collection periods, state the dates for each sample cohort.*
Data exclusions	*If no data were excluded from the analyses, state so OR if data were excluded, provide the exact number of exclusions and the rationale behind them, indicating whether exclusion criteria were pre-established.*
Non-participation	*State how many participants dropped out/declined participation and the reason(s) given OR provide response rate OR state that no participants dropped out/declined participation.*
Randomization	*If participants were not allocated into experimental groups, state so OR describe how participants were allocated to groups, and if allocation was not random, describe how covariates were controlled.*

Ecological, evolutionary & environmental sciences study design

All studies must disclose on these points even when the disclosure is negative.

Study description	*Briefly describe the study. For quantitative data include treatment factors and interactions, design structure (e.g. factorial, nested, hierarchical), nature and number of experimental units and replicates.*

Research sample | *Describe the research sample (e.g. a group of tagged Passer domesticus, all Stenocereus thurberi within Organ Pipe Cactus National*

Research sample | Monument), and provide a rationale for the sample choice. When relevant, describe the organism taxa, source, sex, age range and any manipulations. State what population the sample is meant to represent when applicable. For studies involving existing datasets, describe the data and its source.

Sampling strategy | Note the sampling procedure. Describe the statistical methods that were used to predetermine sample size OR if no sample-size calculation was performed, describe how sample sizes were chosen and provide a rationale for why these sample sizes are sufficient.

Data collection | Describe the data collection procedure, including who recorded the data and how.

Timing and spatial scale | Indicate the start and stop dates of data collection, noting the frequency and periodicity of sampling and providing a rationale for these choices. If there is a gap between collection periods, state the dates for each sample cohort. Specify the spatial scale from which the data are taken

Data exclusions | If no data were excluded from the analyses, state so OR if data were excluded, describe the exclusions and the rationale behind them, indicating whether exclusion criteria were pre-established.

Reproducibility | Describe the measures taken to verify the reproducibility of experimental findings. For each experiment, note whether any attempts to repeat the experiment failed OR state that all attempts to repeat the experiment were successful.

Randomization | Describe how samples/organisms/participants were allocated into groups. If allocation was not random, describe how covariates were controlled. If this is not relevant to your study, explain why.

Blinding | Describe the extent of blinding used during data acquisition and analysis. If blinding was not possible, describe why OR explain why blinding was not relevant to your study.

Did the study involve field work? ☐ Yes ☐ No

Field work, collection and transport

Field conditions | Describe the study conditions for field work, providing relevant parameters (e.g. temperature, rainfall).

Location | State the location of the sampling or experiment, providing relevant parameters (e.g. latitude and longitude, elevation, water depth).

Access & import/export | Describe the efforts you have made to access habitats and to collect and import/export your samples in a responsible manner and in compliance with local, national and international laws, noting any permits that were obtained (give the name of the issuing authority, the date of issue, and any identifying information).

Disturbance | Describe any disturbance caused by the study and how it was minimized.

Reporting for specific materials, systems and methods

We require information from authors about some types of materials, experimental systems and methods used in many studies. Here, indicate whether each material, system or method listed is relevant to your study. If you are not sure if a list item applies to your research, read the appropriate section before selecting a response.

Materials & experimental systems

n/a	Involved in the study	
☐	☒	Antibodies
☐	☒	Eukaryotic cell lines
☒	☐	Palaeontology and archaeology
☐	☒	Animals and other organisms
☒	☐	Human research participants
☒	☐	Clinical data
☐	☒	Dual use research of concern

Methods

n/a	Involved in the study	
☒	☐	ChIP-seq
☒	☐	Flow cytometry
☒	☐	MRI-based neuroimaging

Antibodies

Antibodies used

CSF-1R (clone C-20) Santa Cruz Biotechnology Cat#sc-692
CSF-1R (clone 50059) Sino Biological Cat#50059-T24
F4/80 (clone A3-1) BioRAD Cat#MCA497R
F4/80-AF555 (clone D2S9R) Cell Signaling Technologies Cat#99651BC
Sox9 (clone D8G8H) Cell Signaling Technologies Cat#82630S
Sox9-AF647 (clone D8G8H) Cell Signaling Technologies Cat#71273S
MHC-II (clone M5/114.15.2) Biolegend Cat#110002
CD4 (clone D7D2Z) Cell Signaling Technologies Cat#25229S
CD4-AF555 (clone D7D2Z) Cell Signaling Technologies Cat#25206BC
CD8 (clone 4SM15) eBioscience Cat#13-0808-82
CD3 (clone SP7) Thermo Scientific Cat#MA1-90582
CD3-AF555 (clone D4V8L) Cell Signaling Technologies Cat#72741BC

CD45 (clone 30-F11) BD Pharmingen Cat#550539
CD45-AF555 (clone D3F8Q) Cell Signaling Technologies Cat#19581S
CC3 (clone Asp175) Cell Signaling Technologies Cat#9664S
CC3-AF647 (clone ASP175) Cell Signaling Technologies Cat#9602S
CD45R (clone RA3-6B2) BD Biosciences Cat#550286
CD11c (clone D1V9Y) Cell Signaling Technologies Cat#97585S
CD11c-AF647 (clone D1V9Y) Cell Signaling Technologies Cat#88204BC
CD31 (clone SZ31) Dianova Cat#DIA-310
CD31 (clone EPR17260) Abcam Cat#ab232533
AlphaSMA (clone ab5694) Abcam Cat#ab5694
AlphaSMA-AF488 (clone D4K9N) Cell Signaling Technologies Cat#34105S
Ly6G (clone 1A8) BD Pharmingen Cat#551459
GzmB (clone D6E9W) Cell Signaling Technologies Cat#46890S
GzmB-AF555 (clone D6E9W) Cell Signaling Technologies Cat#90779BC
FoxP3 (clone FJK-16s) Invitrogen Cat#14-5773-82
FoxP3-AF750 (clone NB100-39002) NovusBio Cat#NB100-39002AF750
PD-L1 (clone D5V3B) Cell Signaling Technologies Cat#64988S
PD-L1-AF555 (clone D5V3B) Cell Signaling Technologies Cat#50912BC
Myeloperoxidase (clone Ab-1) Thermo Scientific Cat#Rb-373-A1
Myeloperoxidase (clone 3667) RDSystems Cat#AF3667
PyMT (clone NB100-2749) NovusBio Cat#NB100-2749
MHC-I (clone orb135651) Biorbyt Cat#orb135651
Ki-67 (clone D3B5) Cell Signaling Technologies Cat#9129S
Ki-67-AF647 (clone D3B5) Cell Signaling Technologies Cat#12075S
Galectin-3 (clone M3/38) Biolegend Cat#125402
Galectin-3-AF647 (clone M3/38) Biolegend Cat#125408
Epcam (clone E6V8Y) Cell Signaling Technologies Cat#93790S
Epcam (clone 002) Sino Biological Cat#50591-R002
ICAM-1 (clone 280) Sino Biological Cat#50440-R280
Neuropilin-1 (clone EPR3113) Abcam Cat#ab81321
CD11b (clone EPR1344) Abcam Cat#ab133357
CD11b (clone EPR1344) Abcam Cat#ab21645
CD40 (clone E2Z7J) Cell Signaling Technologies Cat#86165
Arginase-1 (clone D4E3M) Cell Signaling Technologies Cat#93668S
Arginase-1-AF647 (clone D4E3M™) Cell Signaling Technologies Cat#43279S
CD103 (clone 2E7) Biolegned Cat#121402
Axl (clone 854) RDSystems Cat#AF854
Keratin-14-AF647 (clone EPR17350) Abcam Cat#ab206100
Desmin-AF555 (clone Y66) Abcam Cat#ab32362
Collagen-VI (clone EPR17072) Abcam Cat#ab229450
Collagen-IV (clone 203003) MDBiosciences Cat#203003-1
Nf-kB-AF647 (clone D14E12) Cell Signaling Technologies Cat#8801S
Elastin-AF750 (clone C45078) Signalway Antibody Cat#C45078-AF750
MMP-2 (clone EPR1184) Abcam Cat#ab237474
Vimentin-AF647 (clone D21H3) Cell Signaling Technologies Cat#9856S
ESR1 (clone 106132) Sino Biological Cat#106132-T08
E-cadherin (clone 50671) Sino Biological Cat#50671-RP02
Nox-4-AF647 (clone UOTR1B492) Abcam Cat#ab195301
Fibronectin (clone ab45688) Abcam Cat#ab206928
CD56 (clone 108577) Sino Biological Cat#108577-T08
HSP-47 (clone EPR4217) Abcam Cat#ab226052
RIPK1 (clone 106528) Sino Biological Cat#106528-T08
InVivoMAb anti-mouse CSF1R (clone AF598) Bio X Cell Cat#BE0213
InVivoMAb anti-mouse PD-1 (clone RMP1-14) Bio X Cell Cat#BE0146
InVivoMAb anti-mouse CD40 (clone FGK4.5/FGK45) Bio X Cell Cat#BE0016-2
InVivoMAb rat IgG2a isotype control (clone 2A3) Bio X Cell Cat#BE0089

Validation

Antibodies were validated by the vendors for target-specificity. Additionally, all image cytometry analyses performed included samples positively expressing the targets (shown in Extended Data Fig. 1d-f). Multiplex immunohistochemistry specific staining patterns were cross validated against those generated using cyclic immunofluorescence.

Eukaryotic cell lines

Policy information about cell lines

Cell line source(s)

EMT6 cell line was purchased from American Type Culture Collection; E0771 cell line was purchased from CH3 BioSystems®

Authentication	STR by source for the EMT6 cell line; E0771 lot used was not authenticated by CH3 BioSystems®
Mycoplasma contamination	cell were tested negative for Mycoplasma detection
Commonly misidentified lines (See ICLAC register)	no commonly misidentified cell lines were used in this study

Palaeontology and Archaeology

Specimen provenance	*Provide provenance information for specimens and describe permits that were obtained for the work (including the name of the issuing authority, the date of issue, and any identifying information).*
Specimen deposition	*Indicate where the specimens have been deposited to permit free access by other researchers.*
Dating methods	*If new dates are provided, describe how they were obtained (e.g. collection, storage, sample pretreatment and measurement), where they were obtained (i.e. lab name), the calibration program and the protocol for quality assurance OR state that no new dates are provided.*

☐ Tick this box to confirm that the raw and calibrated dates are available in the paper or in Supplementary Information.

Ethics oversight	*Identify the organization(s) that approved or provided guidance on the study protocol, OR state that no ethical approval or guidance was required and explain why not.*

Note that full information on the approval of the study protocol must also be provided in the manuscript.

Animals and other organisms

Policy information about studies involving animals; ARRIVE guidelines recommended for reporting animal research

Laboratory animals	Mice were purchased from The Jackson Laboratory. All mice were bred and housed under specific pathogen free conditions under a standard 12h light / 12h dark cycle. C57LB/6, BALB/c, and FVB/N mice were purchased from the Jackson Laboratory. MMTV-PyMT were from Dr. Lisa Coussens and purchased from the Jackson Laboratory. Virgin female mice of 8-24 weeks of age were used for all experiments.
Wild animals	This study did not involve wild animals
Field-collected samples	This study did not involve samples collected from the field
Ethics oversight	All animal studies were conducted in accordance with protocols approved by Institutional Animal Care and Use Committee (IACUC) at OHSU (protocol number: IP00000956)

Note that full information on the approval of the study protocol must also be provided in the manuscript.

Human research participants

Policy information about studies involving human research participants

Population characteristics	*Describe the covariate-relevant population characteristics of the human research participants (e.g. age, gender, genotypic information, past and current diagnosis and treatment categories). If you filled out the behavioural & social sciences study design questions and have nothing to add here, write "See above."*
Recruitment	*Describe how participants were recruited. Outline any potential self-selection bias or other biases that may be present and how these are likely to impact results.*
Ethics oversight	*Identify the organization(s) that approved the study protocol.*

Note that full information on the approval of the study protocol must also be provided in the manuscript.

Clinical data

Policy information about clinical studies

All manuscripts should comply with the ICMJE guidelines for publication of clinical research and a completed CONSORT checklist must be included with all submissions.

Clinical trial registration	*Provide the trial registration number from ClinicalTrials.gov or an equivalent agency.*
Study protocol	*Note where the full trial protocol can be accessed OR if not available, explain why.*
Data collection	*Describe the settings and locales of data collection, noting the time periods of recruitment and data collection.*
Outcomes	*Describe how you pre-defined primary and secondary outcome measures and how you assessed these measures.*

Dual use research of concern

Policy information about dual use research of concern

Hazards

Could the accidental, deliberate or reckless misuse of agents or technologies generated in the work, or the application of information presented in the manuscript, pose a threat to:

No	Yes	
☒	☐	Public health
☒	☐	National security
☒	☐	Crops and/or livestock
☒	☐	Ecosystems
☒	☐	Any other significant area

Experiments of concern

Does the work involve any of these experiments of concern:

No	Yes	
☒	☐	Demonstrate how to render a vaccine ineffective
☒	☐	Confer resistance to therapeutically useful antibiotics or antiviral agents
☒	☐	Enhance the virulence of a pathogen or render a nonpathogen virulent
☒	☐	Increase transmissibility of a pathogen
☒	☐	Alter the host range of a pathogen
☒	☐	Enable evasion of diagnostic/detection modalities
☒	☐	Enable the weaponization of a biological agent or toxin
☒	☐	Any other potentially harmful combination of experiments and agents

ChIP-seq

Data deposition

☐ Confirm that both raw and final processed data have been deposited in a public database such as GEO.

☐ Confirm that you have deposited or provided access to graph files (e.g. BED files) for the called peaks.

Data access links<br>*May remain private before publication.*	*For "Initial submission" or "Revised version" documents, provide reviewer access links. For your "Final submission" document, provide a link to the deposited data.*
Files in database submission	*Provide a list of all files available in the database submission.*
Genome browser session<br>(e.g. UCSC)	*Provide a link to an anonymized genome browser session for "Initial submission" and "Revised version" documents only, to enable peer review. Write "no longer applicable" for "Final submission" documents.*

Methodology

Replicates	*Describe the experimental replicates, specifying number, type and replicate agreement.*
Sequencing depth	*Describe the sequencing depth for each experiment, providing the total number of reads, uniquely mapped reads, length of reads and whether they were paired- or single-end.*
Antibodies	*Describe the antibodies used for the ChIP-seq experiments; as applicable, provide supplier name, catalog number, clone name, and lot number.*
Peak calling parameters	*Specify the command line program and parameters used for read mapping and peak calling, including the ChIP, control and index files used.*
Data quality	*Describe the methods used to ensure data quality in full detail, including how many peaks are at FDR 5% and above 5-fold enrichment.*
Software	*Describe the software used to collect and analyze the ChIP-seq data. For custom code that has been deposited into a community repository, provide accession details.*

Flow Cytometry

Plots

Confirm that:

- [ ] The axis labels state the marker and fluorochrome used (e.g. CD4-FITC).
- [ ] The axis scales are clearly visible. Include numbers along axes only for bottom left plot of group (a 'group' is an analysis of identical markers).
- [ ] All plots are contour plots with outliers or pseudocolor plots.
- [ ] A numerical value for number of cells or percentage (with statistics) is provided.

Methodology

Sample preparation	*Describe the sample preparation, detailing the biological source of the cells and any tissue processing steps used.*
Instrument	*Identify the instrument used for data collection, specifying make and model number.*
Software	*Describe the software used to collect and analyze the flow cytometry data. For custom code that has been deposited into a community repository, provide accession details.*
Cell population abundance	*Describe the abundance of the relevant cell populations within post-sort fractions, providing details on the purity of the samples and how it was determined.*
Gating strategy	*Describe the gating strategy used for all relevant experiments, specifying the preliminary FSC/SSC gates of the starting cell population, indicating where boundaries between "positive" and "negative" staining cell populations are defined.*

- [ ] Tick this box to confirm that a figure exemplifying the gating strategy is provided in the Supplementary Information.

Magnetic resonance imaging

Experimental design

Design type	*Indicate task or resting state; event-related or block design.*
Design specifications	*Specify the number of blocks, trials or experimental units per session and/or subject, and specify the length of each trial or block (if trials are blocked) and interval between trials.*
Behavioral performance measures	*State number and/or type of variables recorded (e.g. correct button press, response time) and what statistics were used to establish that the subjects were performing the task as expected (e.g. mean, range, and/or standard deviation across subjects).*

Acquisition

Imaging type(s)	*Specify: functional, structural, diffusion, perfusion.*
Field strength	*Specify in Tesla*
Sequence & imaging parameters	*Specify the pulse sequence type (gradient echo, spin echo, etc.), imaging type (EPI, spiral, etc.), field of view, matrix size, slice thickness, orientation and TE/TR/flip angle.*
Area of acquisition	*State whether a whole brain scan was used OR define the area of acquisition, describing how the region was determined.*

Diffusion MRI ☐ Used ☐ Not used

Preprocessing

Preprocessing software	*Provide detail on software version and revision number and on specific parameters (model/functions, brain extraction, segmentation, smoothing kernel size, etc.).*
Normalization	*If data were normalized/standardized, describe the approach(es): specify linear or non-linear and define image types used for transformation OR indicate that data were not normalized and explain rationale for lack of normalization.*
Normalization template	*Describe the template used for normalization/transformation, specifying subject space or group standardized space (e.g. original Talairach, MNI305, ICBM152) OR indicate that the data were not normalized.*
Noise and artifact removal	*Describe your procedure(s) for artifact and structured noise removal, specifying motion parameters, tissue signals and physiological signals (heart rate, respiration).*

Volume censoring | *Define your software and/or method and criteria for volume censoring, and state the extent of such censoring.*

Statistical modeling & inference

Model type and settings | *Specify type (mass univariate, multivariate, RSA, predictive, etc.) and describe essential details of the model at the first and second levels (e.g. fixed, random or mixed effects; drift or auto-correlation).*

Effect(s) tested | *Define precise effect in terms of the task or stimulus conditions instead of psychological concepts and indicate whether ANOVA or factorial designs were used.*

Specify type of analysis: ☐ Whole brain ☐ ROI-based ☐ Both

Statistic type for inference
(See Eklund et al. 2016) | *Specify voxel-wise or cluster-wise and report all relevant parameters for cluster-wise methods.*

Correction | *Describe the type of correction and how it is obtained for multiple comparisons (e.g. FWE, FDR, permutation or Monte Carlo).*

Models & analysis

n/a	Involved in the study
☐	☐ Functional and/or effective connectivity
☐	☐ Graph analysis
☐	☐ Multivariate modeling or predictive analysis

Functional and/or effective connectivity | *Report the measures of dependence used and the model details (e.g. Pearson correlation, partial correlation, mutual information).*

Graph analysis | *Report the dependent variable and connectivity measure, specifying weighted graph or binarized graph, subject- or group-level, and the global and/or node summaries used (e.g. clustering coefficient, efficiency, etc.).*

Multivariate modeling and predictive analysis | *Specify independent variables, features extraction and dimension reduction, model, training and evaluation metrics.*

This checklist template is licensed under a Creative Commons Attribution 4.0 International License, which permits use, sharing, adaptation, distribution and reproduction in any medium or format, as long as you give appropriate credit to the original author(s) and the source, provide a link to the Creative Commons license, and indicate if changes were made. The images or other third party material in this article are included in the article's Creative Commons license, unless indicated otherwise in a credit line to the material. If material is not included in the article's Creative Commons license and your intended use is not permitted by statutory regulation or exceeds the permitted use, you will need to obtain permission directly from the copyright holder. To view a copy of this license, visit http://creativecommons.org/licenses/by/4.0/
